# Tracing growth patterns in cod (*Gadus morhua* L.) using bioenergetic modelling

**DOI:** 10.1002/ece3.10751

**Published:** 2023-11-23

**Authors:** Steffen Funk, Nicole Funk, Jens‐Peter Herrmann, Hans‐Harald Hinrichsen, Uwe Krumme, Christian Möllmann, Axel Temming

**Affiliations:** ^1^ Institute of Marine Ecosystem and Fishery Science, Centre for Earth System Research and Sustainability (CEN) University of Hamburg Hamburg Germany; ^2^ GEOMAR Helmholtz Centre for Ocean Research Kiel Kiel Germany; ^3^ Thünen Institute of Baltic Sea Fisheries Rostock Germany

**Keywords:** Baltic Sea, bioenergetic modelling, climate change, cod, fish growth

## Abstract

Understanding individual growth in commercially exploited fish populations is key to successful stock assessment and informed ecosystem‐based fisheries management. Traditionally, growth rates in marine fish are estimated using otolith age‐readings in combination with age‐length relationships from field samples, or tag‐recapture field experiments. However, for some species, otolith‐based approaches have been proven unreliable and tag‐recapture experiments suffer from high working effort and costs as well as low recapture rates. An important alternative approach for estimating fish growth is represented by bioenergetic modelling which in addition to pure growth estimation can provide valuable insights into the processes leading to temporal growth changes resulting from environmental and related behavioural changes. We here developed an individual‐based bioenergetic model for Western Baltic cod (*Gadus morhua*), traditionally a commercially important fish species that however collapsed recently and likely suffers from climate change effects. Western Baltic cod is an ideal case study for bioenergetic modelling because of recently gained in‐situ process knowledge on spatial distribution and feeding behaviour based on highly resolved data on stomachs and fish distribution. Additionally, physiological processes such as gastric evacuation, consumption, net‐conversion efficiency and metabolic rates have been well studied for cod in laboratory experiments. Our model reliably reproduced seasonal growth patterns observed in the field. Importantly, our bioenergetic modelling approach implementing depth‐use patterns and food intake allowed us to explain the potentially detrimental effect summer heat periods have on the growth of Western Baltic cod that likely will increasingly occur in the future. Hence, our model simulations highlighted a potential mechanism on how warming due to climate change affects the growth of a key species that may apply for similar environments elsewhere.

## Graphical abstract

A bioenergetic model, which includes in‐situ knowledge of distribution and food intake, allows tracing (seasonal) growth patterns of cod. The model shows that summer heat periods can lead to negative energy turnover, ultimately resulting in reduced growth and potentially in a reduced reproductive potential. A detrimental effect that may occur more often in the future in the course of ongoing climate change.

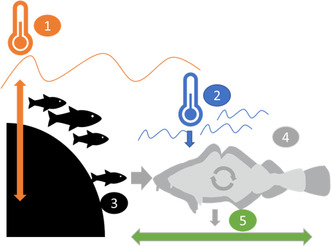

## 1 INTRODUCTION

Understanding individual growth in commercially exploited fish populations is key to successful stock assessment and informed ecosystem‐based fisheries management (Aires‐da‐Silva et al., 2015; Kell & Bromley, 2004; Mion et al., 2021). Traditionally, growth rates in marine fish are estimated using otolith age‐readings in combination with age‐length relationships from field samples. However, for many species and stocks otolith‐based approaches have not been validated (Beamish & McFarlane, 1983) or have been proven unreliable since patterns in otolith ring formation are variable and the driving mechanisms are not well understood (de Pontual et al., 2006; Hüssy et al., 2016). Poor quality of otolith age‐readings challenge the scientific analytical stock assessments and, in extreme cases, may even lead to a suspension of scientific advices, for example, in Eastern Baltic cod (Hüssy et al., 2016; ICES, 2015).

Tag‐recapture experiments are another traditional method for growth rate estimation and are particularly suitable for long‐lived species such as gadoids (McQueen et al., 2019; Piñeiro et al., 2007; Shackell et al., 1997; Tallack, 2009). Disadvantages of this method are, however, high working effort and costs. Furthermore, reliability and quality of growth estimates strongly depend on recapture rates, which often do not reach desirable levels. Recent tag‐recapture experiments on cod in the Western Baltic Sea, for example, resulted in recapture rates of less than 1%, highlighting the challenges of this approach (Krumme et al., 2020).

An important alternative approach for estimating fish growth is represented by bioenergetic modelling (Hansen et al., 1933; Ney, 1993). In addition to pure growth estimation, bioenergetic modelling can provide valuable insights into the processes leading to temporal growth changes resulting from environmental and related behavioural changes (Krohn et al., 1997; Lavaud et al., 2019; van Deurs et al., 2022). Bioenergetic models are generally based on the second law of thermodynamics, described by an energy balance equation. The growth rate of an individual is determined as the difference between the energy uptake via food consumption and the sum of energy output rates including metabolic and waste losses as well as specific dynamic action (Kitchell et al., 1977). During recent decades, bioenergetic models have been widely used to estimate fish growth under controlled (e.g. aquaculture) (e.g. Cuenco et al., 1985) as well as under variable natural conditions (e.g. Beauchamp, 2009; Constantini et al., 2008; Kitchell et al., 1977). The latter requires a sound knowledge on the spatial and temporal distribution of the modelled species and the in situ environmental conditions it experiences. Ambient temperature plays a particularly important role since thermal conditions directly affect physiological processes in poikilothermic animals like most fish species, including energy uptake through consumption and metabolic losses (Temming & Herrmann, 2003; Tirsgaard et al., 2015). Realistic consumption rates and related energy intakes can be estimated based on field data on food intake, including diet compositions and stomach content weights (for cod see e.g. Hansson et al., 1996, Neuenfeldt et al., 2019).

We here developed an individual‐based bioenergetic model for Western Baltic cod (*Gadus morhua*), traditionally a commercially important fish species that, however, collapsed recently (Möllmann et al., 2021). In addition to overfishing, climate change effects are hypothesised to negatively affect cod in the Western Baltic. However, process‐knowledge on how ocean warming has and is affecting important population processes such as growth is largely lacking. Western Baltic cod (WBC) is additionally an ideal case study species for bioenergetic modelling because of recently gained in‐situ process knowledge on spatial distribution (Funk et al., 2020) and feeding behaviour (Funk et al., 2021).

Thereby, Funk et al., 2020 set up a model based on gillnet fishery data which enabled predictions on WBC catch depths (assumingly resembling the preferable residence depth of cod) by prevailing temperature conditions. In further investigations, Funk et al. (2021) were able to shed light in particular on size‐, seasonal‐ and depth‐related patterns in WBC diet compositions. Furthermore, they found a strong relationship between food intake, cod size, ambient water temperature and catch depth.

Additionally, physiological processes such as gastric evacuation (Andersen, 2001; Andersen et al., 2016; dos Santos & Jobling, 1991, 1995; Temming & Andersen, 1994; Temming & Herrmann, 2003; Ursin et al., 1985), consumption (Temming & Herrmann, 2003), net‐conversion efficiency (Temming & Herrmann, 2009) and metabolic rates (Jobling, 1982; Saunders, 1963) have been well studied for cod in laboratory experiments. Hence, there exists a unique data basis for applying a bioenergetic model approach for cod in the Western Baltic.

The availability of reliable length‐at‐age data from international coordinated monitoring surveys (i.e. the Baltic international trawl survey) and contemporary growth estimations based on tag‐recapture studies (McQueen et al., 2019) provide the ideal basis for subsequent validation of the bioenergetic growth model performance. Furthermore, in their growth analyses, McQueen et al. (2019) also discovered clear seasonal patterns in the growth of WBC. However, the extent to which these growth patterns in WBC can be linked to environmental influences such as ambient temperature, related patterns in food intake as well as habitat use remained unclear so far. Here, a bioenergetic model incorporating environmentally driven functions on spatiotemporal distribution, food intake and physiological functions may provide further insights into why and how these seasonal growth patterns emerge.

Hence, in this study, we present an individual‐based bioenergetic growth model of WBC where we explicitly incorporate recently gained ecological process knowledge and aim to discuss the model's reliability and suitability as an additional or even alternative tool to traditional growth estimation methods.

Furthermore, we aim to use the model as a platform to derive further insights into the ecology of WBC by (i) investigating whether seasonal and/or interannual growth patterns of WBC in relation to prevailing temperature conditions emerge, and if so (ii) to discuss how these growth patterns stand in line with those revealed from traditional methods. Eventually, we aim (iii) to put observed growth patterns in relation to stock‐ and area‐specific characteristics including considerations on future changes due to climate warming.

## 2 MATERIALS AND METHODS

### 2.1 Study area

Our bioenergetic model is designed for cod in the Belt Sea, which is part of the Western Baltic Sea (Appendix S1: Figure A1). The Belt Sea is relatively shallow (70% of the area < 20 m) and characterised by a stratified brackish‐water body with only a small tidal range (~10 cm) (Leppäranta & Myrberg, 2000; Snoeijs‐Leijonmalm & Andrén, 2017). Because the Belt Sea lies in a transition zone between the North Sea and the Baltic proper, continuous fluctuations in hydrography are typical which result from wind‐induced inflows of saline bottom water from the north (Kattegat) and surface outflow from the east through the Danish Straits.

### 2.2 Bioenergetic model

**FIGURE 1:**
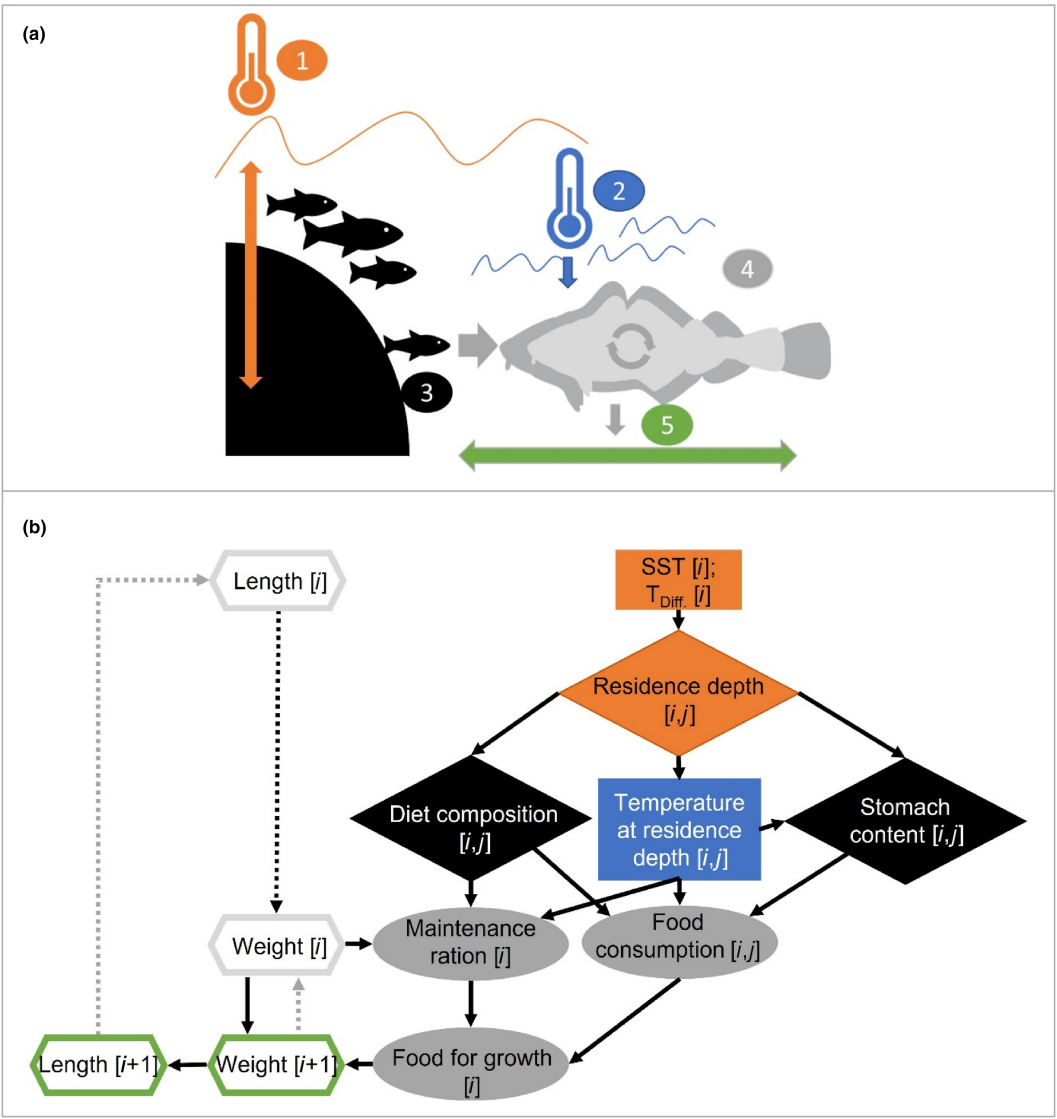
Set‐up of the individual‐based bioenergetic growth model. (a) Schematic representation of important model components and their influence on growth: (1) temperature conditions (SST and *T*_Diff_, see material and methods) (2) temperature at residence depth, (3) food availability and related food intake, (4) physiological processes and (5) growth in length. (b) Functional pathways (arrows) within the bioenergetic model: rectangles denote temperature variables (SST and *T*_Diff_), diamonds denote estimates derived from sub‐models I–III; grey ellipses indicate estimates based on published physiological functions; hexagons display cod individual length and weight. *i*—time step, *j*—size.

Our bioenergetic model represents the growth dynamics of WBC individuals. The model is based on three sub‐models: (i) predicts cod residence depth (i.e. the depth where the cod tend to stay) in relation to ambient water temperature and cod length, (ii) predicts stomach contents in relation to cod length, water temperature and residence depth and (iii) predicts diet composition in relation to cod length, residence depth and season (see Appendices S4 and S9). All three sub‐models were integrated and embedded in cod physiology to predict their combined effect on growth. Our bioenergetic model is thus designed to estimate daily growth of individual cod within a year (Figure 1). We used two successive time periods: 2016–2017 and 2017–2018 (i.e. the periods for which seasonal‐ and regional‐resolved stomach data were available [see Funk et al., 2021]), to assess model performance under two different annual temperature cycles.

Temperature plays a central role in our bioenergetic model since it has a strong influence on the ecology and physiology of poikilothermic animals. More specifically to WBC, we used water temperature to model the residence depth of individual fish for every day within a year (see sub‐model I). Knowing the residence depth of individuals allowed us to derive the ambient temperature each cod is experiencing. The prevailing ambient temperatures are subsequently used to predict their influence on food intake (see sub‐models II & III) and physiology (i.e. consumption and metabolism), together being the main factors influencing cod growth. Ultimately, daily growth of individual cod is estimated by balancing energy intake through consumption and energy loss through metabolism.

The model uses an iterative approach so that the size of individual fish (in length and weight) changes continuously during the modelling period in dependence of its daily growth rate. All parameters that are affected by fish size are adjusted correspondingly, including residence depth, food intake and physiology.

#### 2.2.1 Length and weight at age

We used data on length and weight at age of WBC to provide the initial length for each model run and to calculate length‐weight relationships. Length and weight at age data were derived from monitoring by the Baltic International Trawl Survey (BITS; ICES, 2017, 2019) covering the first quarter of each year during 1991–2018. We used additional data from the Sound (see Appendix S1), that forms together with the Belt Sea the distributional core area of the WBC stock, which allowed us to increase the total number of observations for computing length‐weight relationships. Due to similar hydrographic conditions and a comparable food supply, we assumed no differences in length and weight between the two subareas. Note that we generally assume that survey catches do better reflect the real length distributions in the population for males than for females. Hence, we decided to set up our model for male individuals only (see Appendix S2 for further explanation on this decision). To obtain the initial lengths needed for our model run, we randomly drew n‐fold (in this case 1000) length observations from the BITS Q1 age‐length table. The observations were drawn from the corresponding year (i.e. 2016 or 2017) and age class (i.e. ages 2–4).

#### 2.2.2 Length‐weight relationships

Most of the sub‐models of our bioenergetic model are based on length, while the physiological functions (i.e. metabolism and consumption; see below) are based on weight. Hence, a repeated conversion between length and weight is required during the modelling process. Therefore, we developed two length‐weight relationships (LWR), one for post‐spawning males (LWR_post‐spawning_; *N* = 236; *R* ^2^ = .95) (Equation 1, Table 1), and one for ripe males (i.e. late pre‐spawning and spawning individuals) (LWR_spawning_; *N* = 3512; *R* ^2^ = .98) (Equation 2, Table 1):

(1) $$W_{\text{post‐spawning}}=\alpha_{\text{post‐spawning}}*L^{\beta_{\text{post‐spawning}}}$$

(2) $$W_{\text{spawning}}=\alpha_{\text{spawning}}*L^{\beta_{\text{spawning}}}$$

with $W_{\text{post‐spawning}}$ and $W_{\text{spawning}}$ = weight of post‐spawning and spawning cod [g], *L* = length of cod [cm], and model coefficients $\alpha_{\text{post‐spawning}}$, $\alpha_{\text{spawning}}$, $\beta_{\text{post‐spawning}}$ and $\beta_{\text{spawning}}$.

**TABLE 1:** Overview of equations in the bioenergetic model including parameter coefficient description, unit and values as well literature sources used.

Equation	Parameter	Description	Units	Value	Literature source
1	$\alpha_{\text{post‐spawning}}$	Condition related coefficient	$g\;{\mathrm{cm}}^{-\beta_{\text{post‐spawning}}}$	0.01	Data for calculation taken from BITS Q1 (ICES, 2019)
	$\beta_{\text{post‐spawning}}$	Length exponent	–	2.97	
2	$\alpha_{\text{spawning}}$	Condition related coefficient	$g\;{\mathrm{cm}}^{-\beta_{\text{spawning}}}$	0.008	Data for calculation taken from BITS Q1 (ICES, 2019)
	$\beta_{\text{spawning}}$	Length exponent	–	3.05	
3	$\beta_{0}$	Intercept	m	20.21	Funk et al. (2020)
	$\beta_{1}$	Temperature coefficient	$m{}^{\circ}C^{-1}$	−2.73	
	$\beta_{2}$	Temperature squared coefficient	$m{}^{\circ}C^{-2}$	0.11	
	$\beta_{3}$	*T*_Diff._ coefficient	$m{}^{\circ}C^{-1}$	0.58	
	$f_{j<55\;\mathrm{cm}}$	Factor variable for cod size‐related (i.e. *j*‐related) depth‐use for fish <55 cm	m	0.0	
	$f_{j\geq 55\;\mathrm{cm}}$	Factor variable for cod size‐related (i.e. *j*‐related) depth‐use for fish ≥55 cm	m	3.9	
	ε	Random residual	m	^a^	
4	ρ	Prey‐specific (i.e. *k*‐specific) gastric evacuation coefficient	$g\;g^{-\left(1-\alpha \right)}g^{-\gamma }h^{-1}$	^b^	Temming and Herrmann (2003)
	$\alpha_{1}$	Shape parameter	–	0.5	
	$\gamma_{1}$	Weight exponent	–	0.305	
	$\delta_{1}$	Temperature exponent	${}^{\circ}C^{-1}$	0.11	
5	$\alpha_{2}$	Weight‐specific maintenance rate	$\text{kcal}\;{\mathrm{day}}^{-1}g^{-\gamma }$	0.012	Hansson et al. (1996), Panten (1995), Saunders (1963)
	$\delta_{2}$	Temperature exponent	${}^{\circ}C^{-1}$	0.056	
	$\gamma_{2}$	Weight exponent	–	0.736	
	Act	Activity multiplier	–	1.25	

^a^ Random residual term is randomly drawn for every individual at each time step *i*.

^b^ Prey‐specific gastric evacuation coefficient relates to the diet composition (i.e. diet cluster) and thus varies between individuals at each time step *i* (for further details see Appendix S7).

Since the aim of our study was to model annual growth of adult (i.e. sexually mature) cod, energetic losses due to spawning activity (i.e. the energy loss of reproductive products) should have been considered. However, internal energetic conversion between energy storage (i.e. liver) and gonads, their timing and energy losses over the spawning period are highly complex and have been poorly investigated so far. Hence, we chose a simplified approach and assumed the spawning period to last a single day only and would have occurred before the start of the growth season. This rough model assumption allowed us to start model runs with post‐spawning individuals (all weight losses already considered), which then grow for 365 days, without considering further energy losses due to batch spawning activities or gonad ripening. We chose February 14 (45th day of the year) as the one spawning day, which reflects a time early in the spawning season of Belt Sea cod which lasts from January to June (Bleil et al., 2009; Kändler, 1949; Thurow, 1970). Correspondingly February 15 was set as the starting day for model runs that lasted until February 14 of the following year. The initial start weight of individuals was calculated using the LWR_post‐spawning_ to consider the replenishment of exhausted energy storage capacities after spawning. All further translations from length to weight and vice versa during the modelling were calculated using LWR_spawning_.

Note that continuous translations from weight to length might cause the problem that a loss in weight would have resulted in a decrease in length, which is not reasonable under a physiological point of view. Hence, in these cases, we decided to set $L_{i+1}$ equally to $L_{i}$ (see also subsection Growth below).

#### 2.2.3 Water temperature

Data on water temperature were derived from retrospective simulations with the hydrodynamic Kiel Baltic Sea Ice‐Ocean Model (BSIOM; Lehmann & Hinrichsen, 2000, Lehmann et al., 2002, Lehmann et al., 2014; for further description see Appendix S3). BSIOM provides daily temperature data for 3 m vertical depth strata. We used sea surface temperature (SST) and a proxy for water column stratification (*T*_Diff_) to predict the residence depth of cod (see sub‐model I). *T*_Diff_ was computed as the temperature difference between the depth strata 0–3 m (i.e. SST) and the mean of the depth strata 21–27 m. The latter reflects the bottom temperature in the deeper channels of the Belt Sea. For further calculations (e.g. sub‐model II, physiological functions) ambient water temperatures at residence depths were used.

#### 2.2.4 Sub‐models on cod ecology

##### Residence depth

To predict the daily residence depth of cod a linear regression model (Funk et al., 2020) was used (sub‐model I; see Appendix S4). Originally the model predicts depth selected by Western Baltic gill net fishers (assumed to reflect residence depth of cod) as a function of SST and *T*_Diff_. We slightly modified the original relationship by translating the mesh size factor to a fish size factor, that is larger cod prefer deeper waters than their smaller conspecifics (see Appendices S4 and S5). We accounted for individual variability in the depth‐use of cod by adding a random residual of the depth‐use model on each depth prediction (Equation 3, Table 1):

(3) $$\mathrm{Res}.d.{\;}_{\mathrm{ij}}=\beta_{0}+\beta_{1}*{\mathrm{SST}}_{i}+\beta_{2}*{\mathrm{SST}}_{i}^{2}+\beta_{3}*T_{\text{Diff}.}+f_{j}+\varepsilon_{i}$$

with $\mathrm{Res}.d.{\;}_{\mathrm{ij}}$ = residence depth of a cod of size *j* at time step *i* [m], ${\mathrm{SST}}_{i}$ = sea surface temperature at time step I [°C], $T_{\text{Diff}.}$ = proxy for stratification at time step *i* [°C], $f_{j}$ = depth‐use factor related to cod size j, $\varepsilon_{i}$ = random residual at time step *i*, and model coefficients $\beta_{0}$, $\beta_{1}$ and $\beta_{2}$.

##### Food intake

In our bioenergetic model estimates for food intake of cod are derived from observed stomach content weights. For this purpose, a Generalised Additive Model (GAM; sub‐model II) by Funk et al. (2021) was applied to predict stomach content weights of cod as a function of length, residence depth and quarter (i.e. seasonal intervals) (see Appendices S4 and S6). A random residual of the GAM model was added to the predicted log‐transformed stomach content weights to account for individual variability.

##### Diet composition

A multinomial logistic regression model (sub‐model III; adapted from Funk et al., 2021, see Appendix S4) was used to predict the probabilities for a certain diet composition group (further termed as diet clusters). These diet clusters resulted from a cluster analysis conducted on relative diet composition data of WBC where eight specific diet clusters had been identified, each dominated by one specific prey type (1, other fish; 2, common shore crab; 3, other/unidentified crustaceans; 4, Pleuronectiformes; 5, Peracarida; 6, Molluska; 7, Clupeiformes; 8, Annelida). The multinomial logistic regression model predicts probabilities for these diet clusters in relation to quarter, cod length and residence depth (Funk et al., 2021). In order to allow individual variability in the stomach compositions and thus prevent always selecting the diet cluster with the highest predicted probability, a vector was created for each cod and day *i* containing each diet cluster x times, where x equalled the corresponding predicted rounded probability percentages of each cluster. A diet cluster was then randomly drawn from the vector to be used as the diet cluster for the cod individual at that time step *i*. The information on diet composition is further used to allocate diet cluster specific gastric evacuation coefficients ($\rho_{k}$) and energy contents to stomach content weights (see Appendix S7) needed for estimating consumption and eventually growth.

#### 2.2.5 Physiological functions

##### Daily food consumption

*D*aily consumption was estimated based on modelled stomach content weights (see sub‐model II) and temperature at residence depth (see above) as well as a corresponding gastric evacuation constant *ρ*_*k*_ (Temming & Herrmann, 2003) (Equation 4, Table 1):

(4) $$C_{24\;i}=24\left[h\right]*\rho_{\mathrm{ki}}*{W_{i}}^{\gamma_{1}}*e^{\delta_{1}*T_{i}}*{S_{i}}^{1-\alpha_{1}}$$

with $C_{24\;i}$ = daily consumption at model time step *i* [g], $\rho_{\mathrm{ki}}$ = allocated prey specific gastric evacuation constant for predicted diet cluster *k* at time step *i*, $W_{i}$ = length dependent full weight of individual cod at time step *i* [g], $T_{i}$ = allocated temperature at residence depth of and time step I [°C], and $S_{i}$ = predicted stomach content weight at time step *i* [g], and the model coefficients $\alpha_{1}$, $\gamma_{1}$ and $\delta_{1}$.

In contrast to the original function which uses weight to calculate daily consumption (Temming & Herrmann, 2003), we assumed that gape and stomach sizes primarily influence food intake. Both factors can be considered constant at a given length and do not fluctuate with condition, unlike weight. Hence, we computed consumption using a fixed weight at a given predator length by converting length into weight using the length‐weight regression LWR_spawing_ (Equation 2, Table 1).

##### Daily maintenance ration

Daily maintenance requirements of individual cod were calculated using parameter estimates derived from laboratory experiments investigating routine metabolism of Atlantic cod (Panten, 1995; Saunders, 1963) (Equation 5, Table 1). In contrast to the calculation of daily consumption, we assumed that daily maintenance ration is directly dependent on current fish weight and, hence, used current predator weight at time step *i* ($W_{i}$). Additionally, an activity multiplier (Act) for cod activity in the field of 1.25 was applied (Hansson et al., 1996):

(5) $$R_{\text{maint}\;i}=\alpha_{2}*e^{\delta_{2}*T_{i}}*{W_{i}}^{\gamma_{2}}*\mathrm{Act}$$

with $R_{\text{maint}\;i}$ = maintenance ration of a cod at time step *i* [kcal day^−1^], $T_{i}$ = the allocated temperature at the residence depth of the cod at time step *i* [°C], $W_{i}$ = the weight of the cod at time step *i* [g], Act = the activity multiplier, and model coefficients $\alpha_{2}$, $\delta_{2}$ and $\gamma_{2}$.

Subsequently, information on prey energy density (see Appendix S7) of the predicted diet cluster *k* at each time step *i* was used to transform the daily maintenance ration from kcal in g of ingested food.

##### Growth

Daily growth of individual cod was calculated following the $K_{3}$ approach (Temming & Herrmann, 2009; for further explanation on the $K_{3}$ see Appendix S8), where $K_{3}$ represents a net conversion efficiency integrating the energy losses of faeces, excretion, and specific dynamic action. $K_{3}$ defines the percentage of the food weight that is converted into weight increase of an individual predator after subtracting the maintenance requirements (in weight) from its total food intake (Equation 6):

(6) $${\mathrm{ffg}}_{i}\;\left[g*{\mathrm{day}}^{-1}\right]=C_{24\;i}\;\left[g*{\mathrm{day}}^{-1}\right]-R_{\text{maint}\;i}\left[g*{\mathrm{day}}^{-1}\right]$$

with ${\mathrm{ffg}}_{i}$ = the food for growth at model time step *i*, $C_{24\;i}$ = the daily consumption at model time step *i*, and $R_{\text{maint}\;i}$ = the maintenance ration at model time step *i*.

Subsequently, the derived daily growth increment (i.e. ${\mathrm{ffg}}_{i}$ * $K_{3}$) was added to the current weight of the predator and resulted in the starting weight for the next time step *i* + 1 (Equation 7):

(7) $$W_{i+1}=W_{i}+{\mathrm{ffg}}_{i}*K_{3}$$

with $W_{i+1}$ = the weight of the cod at time step *i* + 1, $W_{i}$ the weight of the cod at time step *i*, ${\mathrm{ffg}}_{i}$ = the food for growth (i.e. the food in weight left after subtracting maintenance requirements in weight) at time step *i*, and $K_{3}$ = the conversion efficiency.

In our model $K_{3}$ was set to a constant value of 0.35 (Appendix S8). Differences in net conversion efficiency depending on the respective diet composition at a given time step were not considered.

The starting length $L_{i+1}$ for the subsequent modelling period was calculated from $W_{i+1}$ by using the LWR_spawning_ (Equation 8). As mentioned above a loss in weight (i.e. $W_{i+1}<W_{i}$) would have resulted in a decrease in length, which is not reasonable under a physiological point of view and was hence prevented by setting $L_{i+1}$ equally to $L_{i}$ (Equation 9):

(8) $$L_{i+1}={\frac{W_{i+1}}{\alpha }}^{\left(\frac{1}{\beta }\right)}$$

(9) $$L_{i+1}=L_{i}\;\text{if}\;L_{i}>{\frac{W_{i+1}}{\alpha }}^{\left(\frac{1}{\beta }\right)}$$

with $L_{i+1}$—length of the cod at time step *i* + 1, $W_{i+1}$—weight of the cod at time step *i* + 1, and $L_{i}$—length of the cod at time step *i*, and model coefficients α and β taken from the LWR_spawning_ (Equation 2, Table 1).

### 2.3 Model validation

We validated our results comparing the modelled terminal fish length after each period to observed length of the corresponding age classes (3–5) recorded in the study area in 2017 and 2018 (i.e. BITS length at age observations). In addition, we compared parameter estimates of the von Bertalanffy Growth Function (VBGF) derived from our bioenergetic model with those derived from a contemporary tag‐recapture study (McQueen et al., 2019). VBGF parameters *k* and $L_{\infty }$ were calculated using the initial and predicted end lengths of both model periods and all three age classes (Equations 10 and 11). For this purpose, all 6000 individual model estimates were pooled and used in a linear regression of annual length growth increments explained by their initial lengths. The resulting estimates of intercept and slope were used as VBGF parameters *E* and −k, respectively (10) (Gulland, 1969):

(10) $$\frac{∆L}{∆t}=E-k*L$$

(11) $$L_{\infty }=\frac{E}{k}$$

with $\frac{∆L}{∆t}$ = the change in total length of cod ∆L over the time ∆t, *E* = reflecting the hypothetical maximum growth increment for a total length of 0 cm, *k* = the VBGF growth parameter, *L* = the start length of the cod, and $L_{\infty }$ = the hypothetical maximum length of the cod.

### 2.4 Software

All calculations and visualisations were set up and performed in the statistical software and programming environment R (R Development Core Team, 2017), using the packages *plyr* (Wickham, 2011), *MALDIquant*, (Gibb & Strimmer, 2012), *ggplot2* (Wickham, 2009), *cowplot* (Wilke, 2017), *mgcv* (Wood, 2011) and *nnet* (Venables & Ripley, 2002).

## 3 RESULTS

### 3.1 Model validation

**FIGURE 2:**
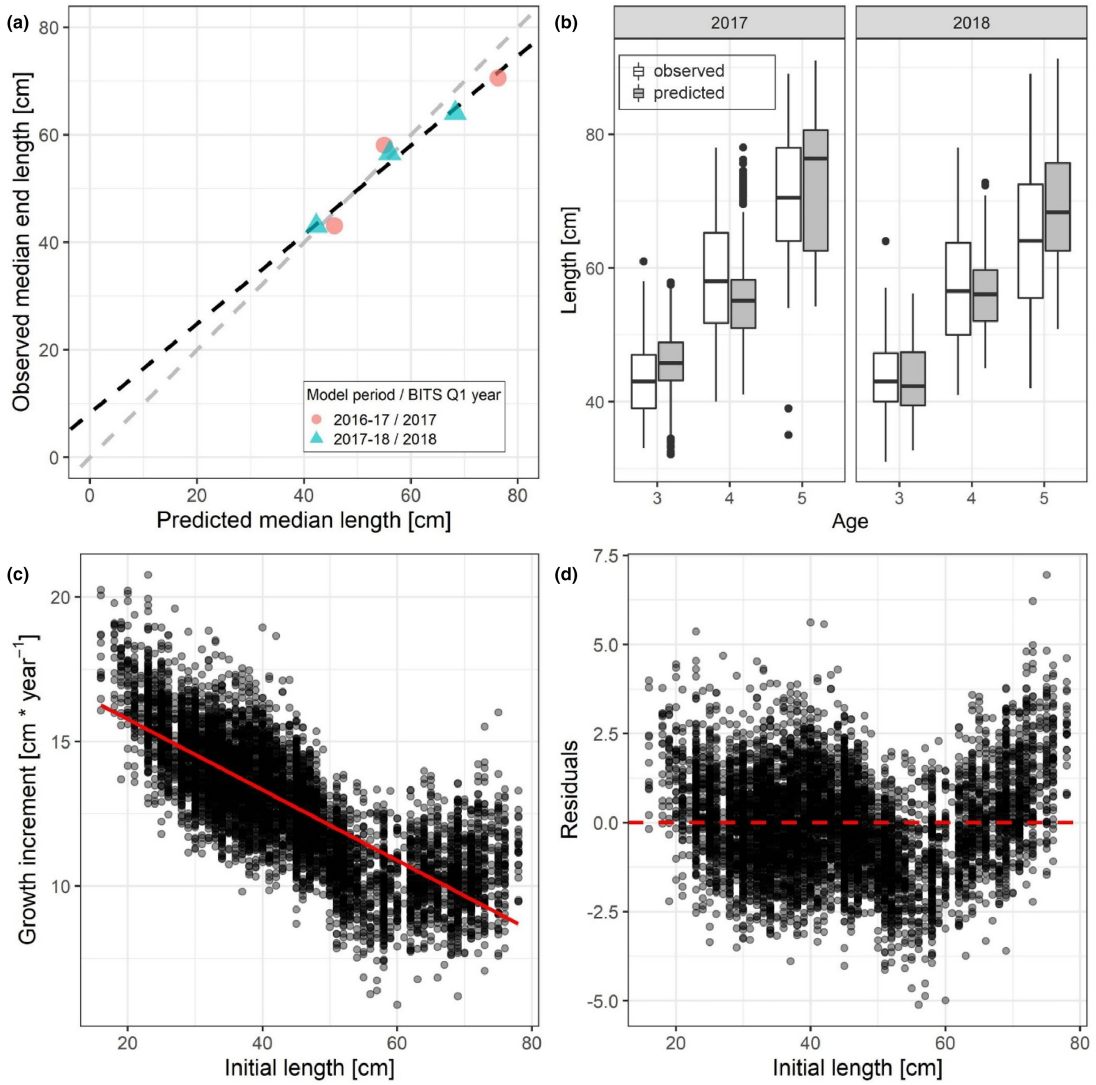
Linear regression between predicted and observed median lengths of Western Baltic cod (a) and linear regression between initial length (start length), boxplots of observed and predicted length (b), and yearly length increments (c) with corresponding regression residuals versus initial length (d). Data in (a) are from the two modelling periods 2016–2017/2017–2018 and corresponding field observations. The dashed grey line in (a) indicates a perfect regression with slope of 1 through the origin, while the dashed black line represents the linear regression model. Boxplots in (b) display the variation in length of both BITS observations (white) and end‐length of cod resulting from the model runs (grey) per age class (note that in case of the model data the displayed age class correspond to start age + 1) and year of the corresponding Q1 BITS. Boxes display median and 25% and 75% quantiles (i.e. lower and upper hinge of boxes). Whiskers range from the upper/lower hinge to the largest value, but no further than 1.5 * the interquartile range (IQR) from the hinge, respectively. Black dots represent outliers above or below 1.5 * IQR from the upper/lower hinge. The solid red line in (c) represents a linear regression model fitted to the data. The dashed red line in (d) depicts the zero line.

We validated our model by comparing predicted mean lengths and model derived parameters for the VBGF to observed data. Predicted median end lengths of WBC (i.e. length after the 365 days modelling cycle) were significantly related to observed median lengths from field observations during the first quarters of years 2017 and 2018 (Figure 2a,b). A linear regression (intercept = 8.22, slope = 0.83) of observed and predicted median lengths (*p* < .01, adjusted *R* ^2^ = .94) revealed only a slight overestimation of predicted lengths by the bioenergetic growth model.

Initial lengths and yearly length increments from the model displayed a significant negative relationship (*p* < .001, *R* ^2^ = .57; Figure 2c). The VBGF parameters (*k* = 0.12 and $L_{\infty }$ = 149.59 cm) resulting from this linear regression (slope = −0.12, Intercept = 18.21) closely resembled observed values (*k* = 0.11 and $L_{\infty }$ = 154.66 cm) (McQueen et al., 2019). The linear model underestimates the growth increments for the smallest and largest starting lengths, whereas the growth for intermediate starting lengths between 50 and 57 cm were slightly overestimated (Figure 2c,d).

### 3.2 Seasonal dynamics in temperature and residence depth

**FIGURE 3:**
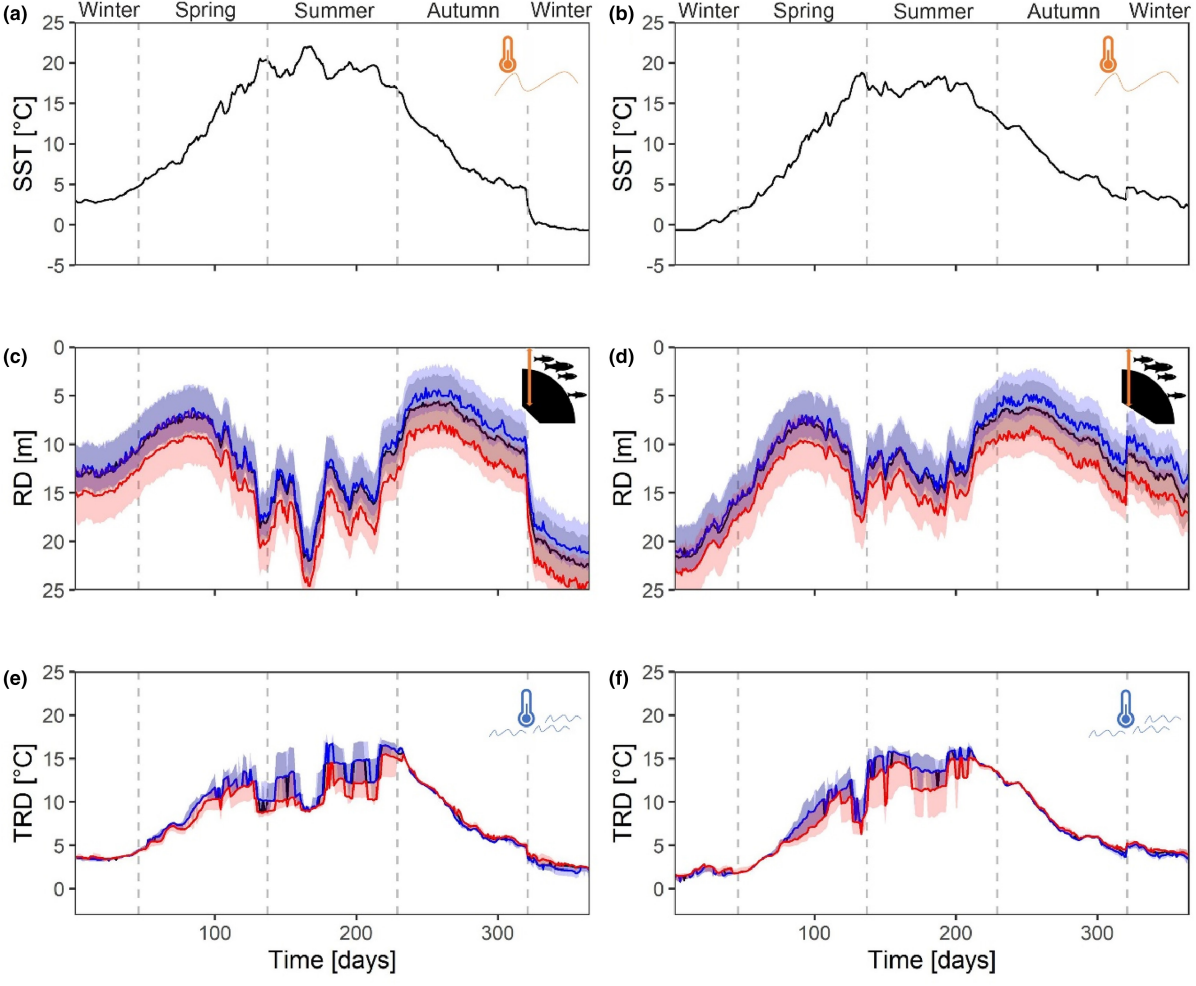
Seasonal dynamics in temperature and residence depth. Sea surface temperature (SST; a, b), residence depth (RD) of cod (c, d), and ambient temperature at residence depths (TRD; e, f). Displayed data represent two modelling periods, that is 2016–2017 (left panels) and 2017–2018 (right panels). RD and TRD are shown for cod age classes 2 (blue), 3 (black) and 4 (red). Solid lines represent medians and shaded areas in c–f show the 25% and 75% quantiles.

Our modelling study shows that the preferred residence depth of WBC during a year which is related to the seasonal dynamics of SST (Figure 3a,b) and stratification of the water column, results in a typical m‐shaped movement pattern (Figure 3c,d). Cod individuals resided in deeper depths (>15 m) during winter and summer (at highest and lowest SST), but in shallower depths (<12 m) during spring and autumn (at medium SST). However, the actual residence depth varied depending on size and age of cod with older individuals staying generally deeper (Figure 3c,d). The seasonal course of the ambient temperatures experienced by cod corresponded to the seasonal dynamics of SST (Figure 3e,f). Highest ambient temperatures (median > 12°C) were experienced by cod during peak summer periods and the lowest during winter periods (median < 5°C).

### 3.3 Seasonality in consumption, maintenance and food for growth

**FIGURE 4:**
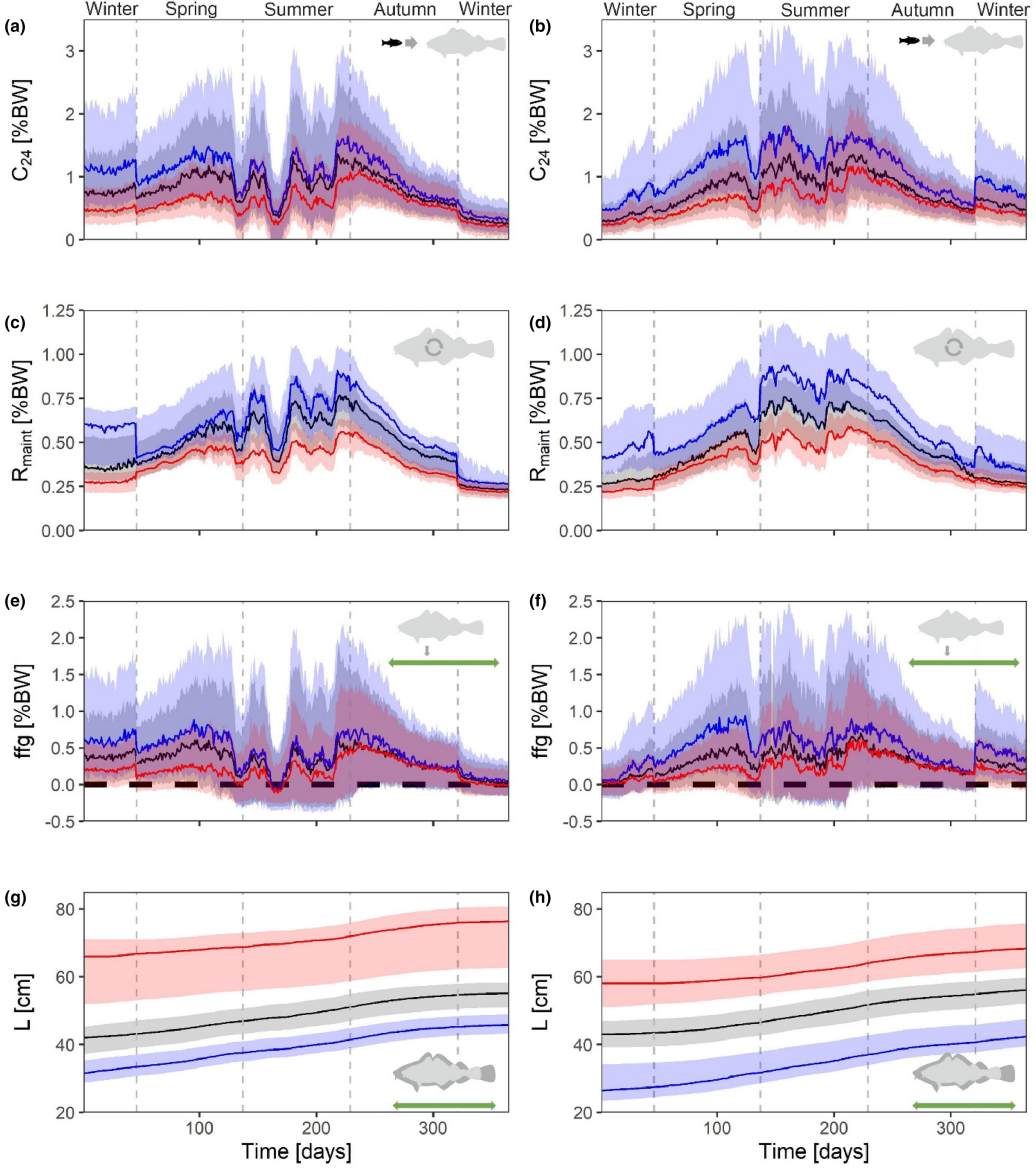
Seasonal development in daily consumption (C_24_) (a, b), daily maintenance ration (*R*_maint_) (c, d) and food for growth (ffg) (e, f), all in % body weight (%BW), as well as length (*L*) (g, h). Displayed data represent two modelling periods, that is 2016–2017 (left panels) and 2017–2018 (right panels). All variables are shown for cod age classes 2 (blue), 3 (black) and 4 (red); solid lines represent medians and shaded areas show the 25% and 75% quantiles; zero lines (e, f) are dashed.

Our model runs demonstrated that rates in consumption and maintenance, and eventually food for growth closely depended on ambient temperatures that are a result of the seasonal residence depth. During shallow water phases daily consumption (C_24_) was highest in all cod ages during both modelling periods. During peak summer, however, when cod reside in deeper waters, daily consumption was lowest (Figure 4a,b). Especially for summer of 2016, the model simulated extremely low C_24_ values (25% quantile of C_24_ extending to zero; Figure 4a). Similarly, seasonal maintenance dynamics (*R*_maint_) resembled those of the ambient temperatures at cod residence depths, with highest metabolic rates during phases of highest ambient temperatures (Figure 4c,d).

Seasonal estimates of food for growth (ffg) followed closely the pattern in consumption. In both years negative ffg estimates indicated periods of weight loss during peak summer, when cod reside in deeper areas (>15 m) and when low consumption co‐occurred at high ambient water temperatures, reflecting high metabolic costs (Figure 4e,f). Length growth of cod strictly followed the seasonal ffg dynamics. Strongest increases in length occurred during phases with high ffg values but flattened during phases of low ffg. Weight losses (i.e. negative food for growth) occurred during peak summer periods (Figure 4g,h).

### 3.4 Interannual differences in growth dynamics

**FIGURE 5:**
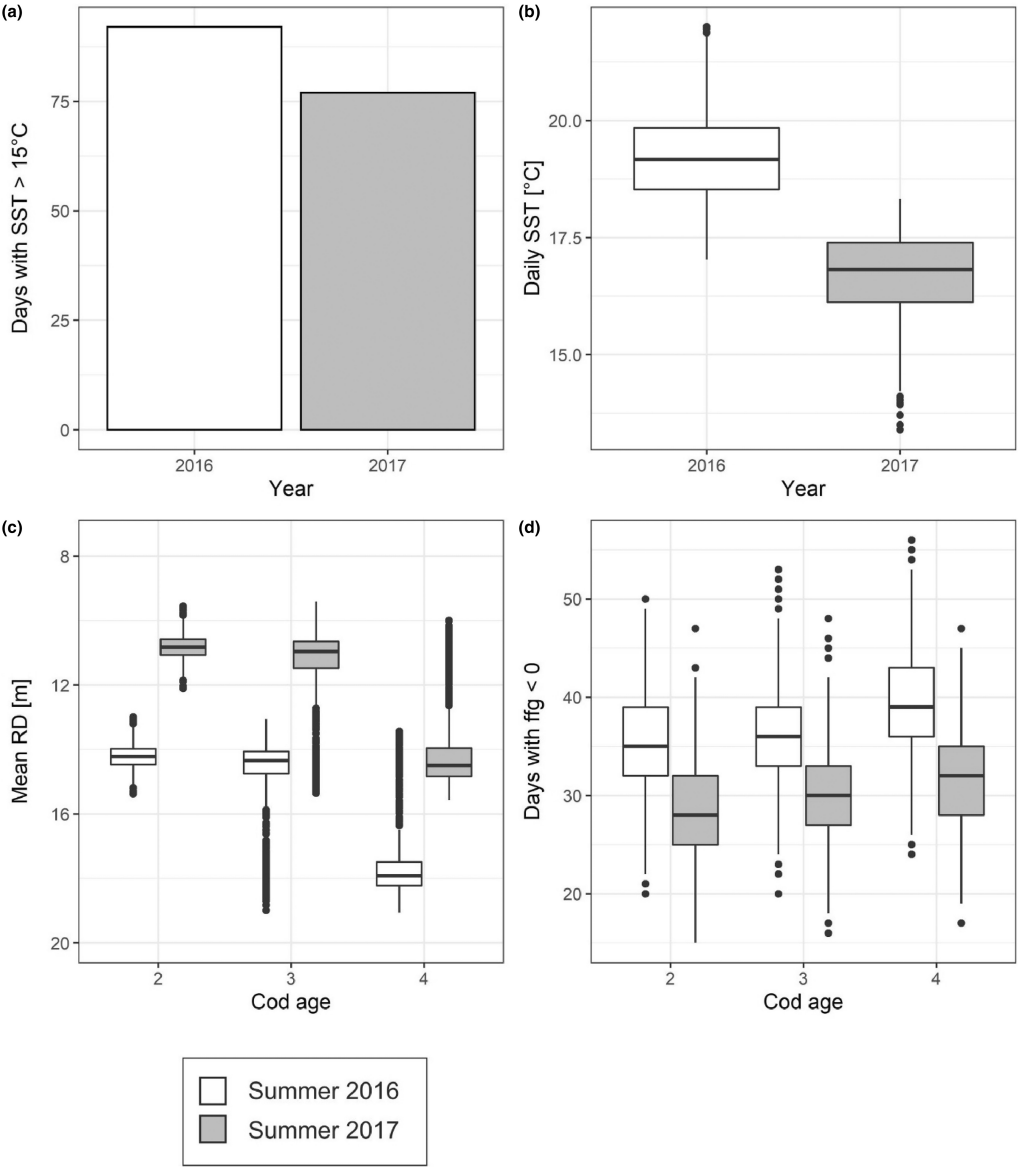
Interannual differences between summer periods in 2016–17 and 2017–18 in (a) number of days with sea surface temperature (SST) >15°C, (b) daily SST (RD), (c) mean residence depth (RD), and (d) number of days with negative food for growth (ffg) values per cod individuals and age class. Boxplots display median and 25% and 75% quantiles (i.e. lower and upper hinge of boxes). Whiskers range from the upper/lower hinge to the largest value, but no further than 1.5 * the interquartile range (IQR) from the hinge, respectively. Black dots represent outliers above or below 1.5 * IQR from the upper/lower hinge.

Our bioenergetic modelling study revealed that growth dynamics of WBC were similar between the periods 2016–17 and 2017–18, because of similar seasonal temperature dynamics (Figure 5e–h). However, interannual differences in growth were obvious during summer. During summer 2016–17, incidences of SST >15°C were observed more frequently (92 days) compared to 2017–18 (77 days) (Figure 5a). Furthermore, SST was on average higher in 2016–17 (19.23 ± 1.26°C) compared to 2017–18 (16.56 ± 1.24°C) (Figure 5b). These differences in thermal regime resulted in a longer over‐summering period in 2016, where cod individuals resided longer in deeper waters (Figure 5c). Longer periods in deeper waters caused lower consumption, reduced daily ffg values and on average lower daily growth increments. The effect is most obvious in the total number of days with negative individual ffg being significantly higher in summer 2016 compared to summer 2017 (Mann–Whitney‐*U*‐test, *p* < .05) (Figure 5d).

## 4 DISCUSSION

### 4.1 Model performance

In this study we present an individual‐based bioenergetic model as a valuable addition to conventional growth estimation approaches such as otolith age‐readings or tag‐recapture experiments. Differences in growth estimates between our model and the BITS Q1 field observations were only minor and mostly apparent for older and larger individuals. A potential explanation for this difference might be size‐selective fishing causing the size distribution to shift towards smaller and slower growing fish in the field (Lee, 1912, for cod see Kristiansen & Svåsand, 1998). However, an overestimation of growth in larger cod in our model can also not be excluded. For example, our estimated metabolic costs based on laboratory studies by Panten (1995) are rather low for larger individuals compared to other estimates, for example, by Jobling (1982). Thus, we might overestimate the overall positive energetic turnover and resulting growth for larger cod individuals by underestimating metabolic requirements. Furthermore, energetic losses through spawning activity, as considered in our model, may not take into account all the energetic costs occurring through spawning activity in the field. Our simplified approach assumes that all energetic costs can be calculated using the differences in weight between a cod before and after spawning. Nevertheless, due to ongoing feeding activity during spawning, we assume that energetic requirements of the overall spawning are likely covered and thus may mitigate the overall weight losses occurring during spawning. The general reliability of model‐based growth estimates is furthermore shown by model‐derived VBGF parameters that were close to recently published estimates based on field data by McQueen et al. (2019). However, the residuals of a linear regression between growth increments and start length, especially at lower (<25 cm) and larger (>60 cm) sizes, suggest an under‐ and overestimation of growth by our VBGF, respectively. Underestimation by the VBGF at lower cod sizes may reflect the limited size range of individuals used for the calculation of sub‐models of stomach content weight and diet composition (only individuals ≥31 cm used; Funk et al., 2021). Moreover, we assumed that all individuals in age class 2 already took part in spawning activity. In fact, the maturity ogive of cod in age class 2 in males was observed to be 86% and 50% in the years 2016 and 2017, respectively (calculated from BITS Q1 data). This indicates that a considerable proportion of males in age class 2 are not taking part in the spawning activity. We thus may slightly underestimate the growth in the model for this age class since the needed energy for spawning might be used for growth instead. The observed pattern in residuals at larger start length (>60 cm) indicates a general trend towards an underestimation of growth by the VBGF for WBC at larger sizes also reported for field data (McQueen et al., 2019). In our model this phenomenon is caused by a change in diet composition of large cod to a greater probability of feeding on flatfishes (Funk et al., 2021). Lean flatfishes are evacuated faster than chitinous crustacean organisms (Andersen et al., 2016; Temming & Herrmann, 2003) and are additionally offering higher prey‐specific energy densities. Therefore, for the same amount of stomach content, a diet dominated by flatfish would lead to significantly higher consumption, energy intake and growth than a crab‐based diet. Such ontogenetic diet shifts, not accounted for in the classical VBGF, leading to increased growth rates of larger individuals, have already been reported for other species, for example, *Osmerus eperlanus* (Vinni et al., 2004).

### 4.2 Model assumptions

Our modelling approach is based on several assumptions that are due to data limitations on bioenergetics of WBC. For example, gastric evacuation of cod is a relatively well‐studied physiological process (Andersen, 2001; Andersen et al., 2016; dos Santos & Jobling, 1991, 1995; Jobling, 1982; Saunders, 1963; Temming & Andersen, 1994; Temming & Herrmann, 2003, 2009). However, gastric evacuation coefficients are not available for all of the major prey species of WBC (e.g. *Carcinus maenas*) and hence had to be taken from species we considered similar (see Appendix S7). Similarly, net conversion efficiency is theoretically different between prey species (see e.g. Temming & Herrmann, 2009) because of their specific composition of proteins, lipids and carbohydrates (Klinger, 2017). Species‐specific conversion efficiency estimates for Atlantic cod prey species are available for brown shrimp (*Crangon crangon*), gobies (*Pomatoschistus* sp.) and smelt (*Osmerus eperlanus*) (Temming, 1995), as well as herring (*Clupea harengus*) and sprat (*Sprattus sprattus*) (Klinger, 2017). However, for some of the main prey species of WBC such as the shore crab (*Carcinus maneas*) no information is available and additionally there is a lack of knowledge on conversion efficiency of mixed diets that are considered higher than single species diets (Klinger, 2017). Due to this lack on comprehensive prey species‐specific information, we applied a rather simplified approach with a constant conversion efficiency of 0.35 for all prey types.

Another limitation of our bioenergetic model is related to the use of the $K_{3}$ concept. $K_{3}$ describes the efficiency of the conversion of ingested prey weight into weight gain of the predator, while no distinction is made about the pathways of the ingested energy (Temming & Herrmann, 2009). Experimental studies with whiting (*Merlangius merlangus*) and saithe (*Pollachius virens*) demonstrated that energy allocation is related to ontogeny and maturity (Andersen & Riis‐Vestergaard, 2003). Two major types of energy allocation are known; the predator either builds up muscle tissue (i.e. somatic growth) or stores fat in its liver (i.e. internal energy store). However, little is known on how ingested energy is allocated (i.e. quantitative distribution) and to what extent these allocation processes are related to food composition (i.e. fat, proteins and carbohydrates). It is furthermore unclear, to what extent somatic growth is limited at the upper‐end of its range for a certain amount of ingested energy and whether a possible energy surplus could always be stored in the liver (as fat reserves). In addition, it is unclear whether, at low consumption on high‐protein but low‐fat prey, cod individuals may use energy from their internal storages (i.e. from the breakdown of liver fats) to cover metabolic costs rather than breaking down energy‐poorer proteins of the ingested prey to support somatic growth like discussed in a recent study by van Deurs et al. (2022). Due to these knowledge deficiencies about energy allocation processes, we here assumed a constant allocation in somatic growth and energy storage reflected by the $K_{3}$ approach.

### 4.3 Novel insights on seasonal and interannual growth patterns

A key feature of our bioenergetic model is the implementation of a depth‐use sub‐model that allowed us to show that shallow‐water habitats (<10 m depths) are critical for WBC growth (Funk et al., 2020), confirming largest daily length increments in autumn reported by a tagging study (McQueen et al., 2019). In our model these high growth rates result directly from high food intake in shallow water due to high prey availability and feeding rates. High food intake during this critical time of the year serves refilling exhausted energy reserves after longer food shortage at their deeper over‐summering habitats needed for maturation processes during the upcoming winter and spawning season (Funk et al., 2020, 2021).

Our estimate of the duration of the shallow water phase might be overestimated since the depth‐use in the bioenergetic model is only related to thermal conditions.

Besides temperature also other abiotic factors such as salinity and oxygen can be considered important drivers influencing the depth‐use of WBC. For example, higher salinities >20 PSU mostly occur in the deeper channels (>20 m depth) of the Western Baltic Sea only. These salinities are essential for successful egg fertilisation and egg buoyancy (Bleil & Oeberst, 2000, 2002; Nissling & Westin, 1997; Petereit et al., 2014). In addition, temporal hypoxic zones may limit the depth and habitat use of WBCs (Receveur et al., 2022).

The spawning behaviour of cod individuals may also play a role in depth selection and duration of stay at these depths. For example, male cod tend to reside and repeatedly spawn at the spawning sites for several weeks during spawning season, while females leave the spawning sites quickly after releasing their eggs (Morgan & Trippel, 1996). However, such a behavioural depth‐use component during the WBC spawning season was not considered in our model. Since in our model the depth‐related movement is entirely triggered by temperature, the high SSTs in winter 2016 may have artificially prolonged the shallow water period resulting in overestimated consumption rates during pre‐spawning and spawning period when food intake is reduced in cod (Fordham & Trippel, 1999). However, in nature, movements of WBC to deeper, more saline water during spawning time might be also triggered by an internal clock or day‐length.

Outside the spawning season, it can be expected that habitat and related depth‐use selection of cod serves three main purposes, namely: maximisation of food supply, shelter and thermoregulation (Freitas et al., 2016; Funk et al., 2020).

Consequently, considering water temperature and cod sizes as the only factors influencing depth‐use of WBC, our model greatly simplifies the numerous drivers and their interactions shaping the actual seasonal depth‐use of WBC in the field. Nonetheless, the analyses of Funk et al. (2020) strongly suggest that direct and indirect water temperature effects (e.g. effects on prey and related movements of WBC to follow up the prey) can be assumed the main drivers of the WBC depth‐use in the area.

Our model predicts reduced growth and occasional weight losses of WBC during summer. Implementing a depth‐use component in the bioenergetic model demonstrated that cod avoid warm surface water layers in summer and move to deeper and colder water layers where less prey is available. Consequently, stomach contents and total consumption and eventually growth are reduced. When SSTs approach extreme (~20°C) levels our model frequently predicted the use of depth >20 m where ambient temperatures reach values around 10°C. By moving to deeper areas, cod can reduce its metabolic requirements. With increasing depth also the food intake of cod predicted by our model decreased severely. A phenomenon that is even more pronounced during summer than in winter, and which could be most likely attributed to spatiotemporal distribution patterns of cod prey organisms (Funk et al., 2021). Although metabolic requirements are reduced in these deep over‐summering habitats, food intake is even so limited, that metabolic costs of cod exceed energy intake rates. This ultimately results in weight loss of individual fish and in general reduced growth. WBC hence face a trade‐off between avoiding high surface temperatures and lower prey availability in deeper waters (Funk et al., 2020). However, the cod may slow down its activity as a result of food shortage at its over‐summering habitats. Lowering activity may allow cod to reduce metabolic costs, a mechanism not considered in our model and which may have slightly exaggerated weight losses in summer. On the other hand, weight losses during summer might be underestimated by the bioenergetic model since the occurrence of hypoxic zones in the deep channels and basins of the Belt Sea (Karlson et al., 2002; Weigelt, 1987) may prevent the downward movement of cod individuals, forcing them to stay in unfavourable shallower areas at high ambient water temperatures. Note that since we used a rather low‐weight exponent in the formula of the maintenance ratio taken from Panten (1995), the resulting calculated negative metabolic turnover might be seen as a rather conservative estimate for larger individuals. Considering higher metabolic losses (e.g. following estimates by Jobling, 1982) would have resulted in even higher negative metabolic turnovers during summer periods.

In general, our bioenergetic modelling approach implementing depth‐use patterns and related food intake allowed us to explain the potentially detrimental effect summer heat periods have on growth of WBC that likely will increasingly occur in the future. The weight loss of cod during summer is a phenomenon which needs further investigation in the field, since it can be an emerging warning signal for the future development of this fish stock under climate change. Longer and stronger summer heatwaves are a typical effect of climate change in the world ocean (Cheung et al., 2021; Frölicher & Laufkötter, 2018) and also in the Baltic Sea (Goebeler et al., 2022). Weight loss during summer that emerged from the bioenergetic model showed that the elevated water temperatures constitute a metabolic bottleneck for WBC. This, in turn, confirms that the translucent zone formed in otoliths during the summer period (Krumme et al., 2020) indeed reflects a period of metabolic stress in WBC. First evidence of weight losses of WBC during heat waves have been reported by local gillnet fishers, who have observed cod in bad nutritional condition (pers. comm. from local gillnet fishers). Consideration of long‐term data series on monthly liver weights and Fulton condition indices of WBC from the Thuenen Institute of Baltic Sea Fisheries (collected as part of the EU Data collective framework (DCF)), also supports the hypothesis of these energetic bottlenecks for WBC during the summer period, with minima not being observed shortly after spawning, as usually observed in other cod stocks (e.g. see Eliassen & Vahl, 1982; Lambert & Dutil, 1997; Schwalme & Chouinard, 1999), but mostly during the summer (see Appendix S10). Weight losses during summer may lead to an overall decreased condition, a reduced growth rate and a decreased build‐up of reproductive products for the upcoming spawning season (Kjesbu et al., 1991; Lambert & Dutil, 2000). Especially the latter may have drastic consequences for the reproductive success of WBC, which can be already considered to be in a critical state (Möllmann et al., 2021; Receveur et al., 2022).

Overall, our model thus illustrates the detrimental effects summer heat periods can have on important key species such as WBC, which is considered one of the ecosystem's apex predators. Similar mechanisms are also conceivable for other ecosystems with similar environmental conditions and for other species or stocks that also occur at the edges of their thermal tolerance limits.

## 5 CONCLUSIONS

Our individual‐based bioenergetic modelling approach demonstrates the importance of a holistic understanding of seasonal variability in habitat use and food intake for the reliable prediction of growth in fish populations. Given such detailed information is available, bioenergetic modelling can be a suitable alternative for growth estimations especially for those species where traditional methods are considered unreliable, for example, otolith‐based estimates in Eastern Baltic cod ( Hüssy et al., 2016).

Besides reliable growth estimations, the bioenergetic modelling is further able to provide insights into driving forces behind seasonal or interannual growth patterns. For example, these insights can detect critical bottleneck phases such as the temperature‐stress‐induced weight loss observed in summer for WBC. These critical growth periods may negatively affect the overall growth dynamics and consequently the reproductive potential of a population. An early identification and mechanistic understanding of these bottlenecks and the related driving processes are hence of crucial importance.

## AUTHOR CONTRIBUTIONS

**Steffen Funk:** Conceptualization (equal); data curation (lead); formal analysis (lead); methodology (equal); resources (equal); software (lead); visualization (lead); writing – original draft (lead); writing – review and editing (equal). **Nicole Funk:** Data curation (supporting); visualization (supporting); writing – review and editing (equal). **Jens‐Peter Herrmann:** Conceptualization (equal); formal analysis (supporting); methodology (equal); writing – original draft (supporting); writing – review and editing (supporting). **Hans‐Harald Hinrichsen:** Resources (equal); writing – original draft (supporting); writing – review and editing (equal). **Uwe Krumme:** Conceptualization (supporting); supervision (equal); writing – review and editing (equal). **Christian Möllmann:** Conceptualization (supporting); formal analysis (supporting); funding acquisition (lead); methodology (equal); supervision (equal); writing – review and editing (equal). **Axel Temming:** Conceptualization (equal); formal analysis (supporting); methodology (equal); supervision (lead); writing – original draft (supporting); writing – review and editing (equal).

## FUNDING INFORMATION

This study received financial support by the Federal Ministry of Education and Research (BMBF) of Germany in the framework of the project *SpaCeParti* (Coastal Fishery, Biodiversity, Spatial Use and Climate Change: A Participative Approach to navigate the Western Baltic Sea into a Sustainable Future; Grant no. 03F0914). Further funding was received for NF from the BMBF‐funded project *balt_ADAPT* (Adaptation of the Western Baltic Coastal Fishery to Climate Change; Grant no. 03F0863D).

## CONFLICT OF INTEREST STATEMENT

All authors have no conflict of interest.

## Supporting information


Appendix S1

## Data Availability

All R codes and data for running the bioenergetic model for Western Baltic cod can be free accessed and downloaded from the open‐access data repository Dryad (Link to repository: https://doi.org/10.5061/dryad.stqjq2c8r).
