# Supplementary material for: Tracing growth patterns in cod (Gadus morhua L.) using bioenergetic modelling
Source: Ecol Evol. 2023 Nov 23;13(11):e10751. doi: 10.1002/ece3.10751 (PMC10667610; doi:10.1002/ece3.10751)
Supplement: Supplementary file 1 — Appendix S1 [file ECE3-13-e10751-s001.docx]

**APPENDIX**

**Appendix S1: Study area**


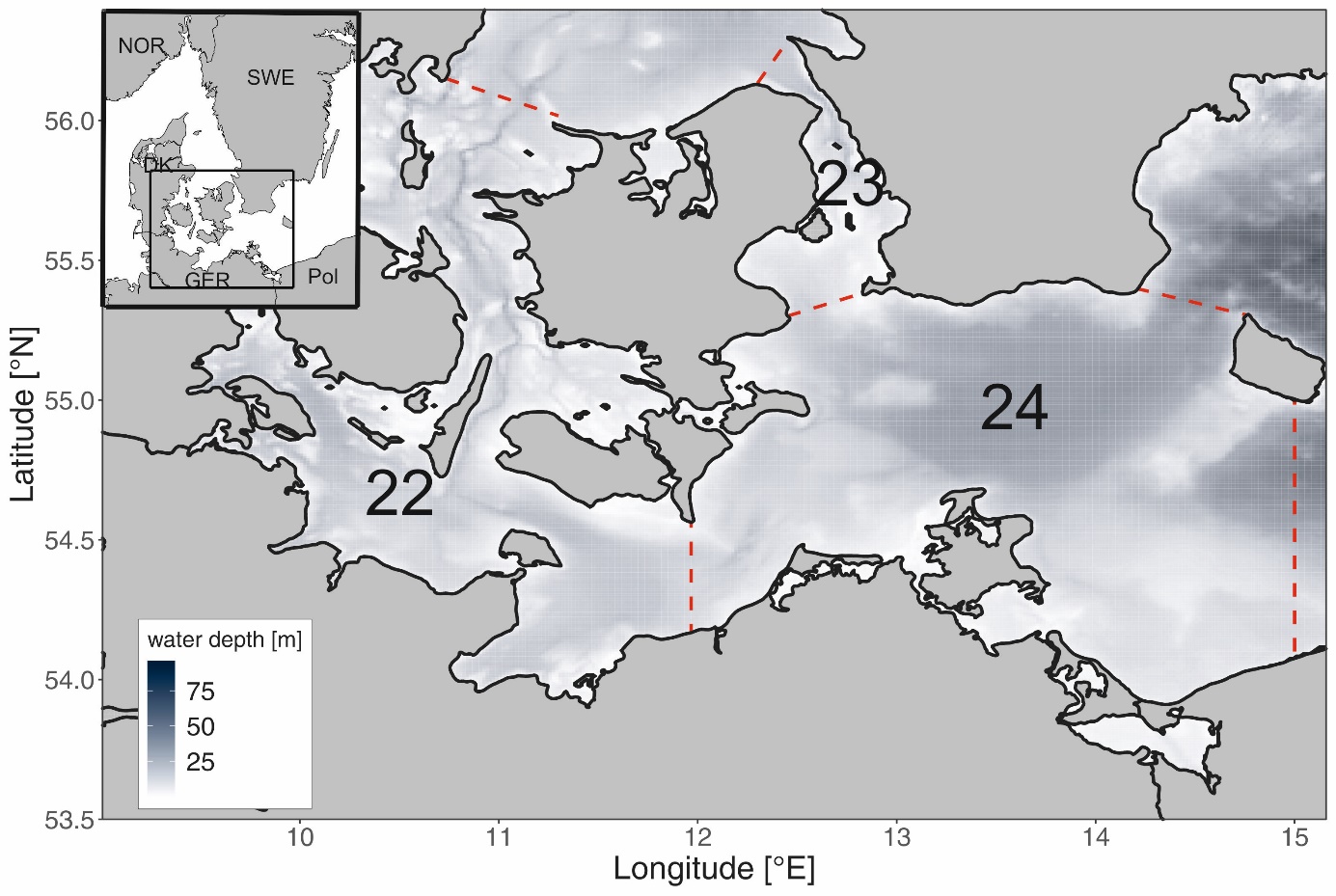


**Appendix Fig. A1.** Bathymetric map of the Western Baltic Sea. The dashed red lines indicate the borders of the subdivisions, which form the Western Baltic Sea: The Belt Sea (SD22; the chosen study area), the Sound (SD23), and the Arkona Sea (SD24).

**Appendix S2: Assumed sex-related differences in the catch composition of BITS hauls**

Sex-specific differences in growth and maturity are common in gadoid species and are also reported for Atlantic cod. Females grow faster, while males often reach maturity earlier in age and size. Moreover, sampling of international trawl surveys (e.g., BITS) take place mostly in deep trawlable areas, which are used by cod mostly for spawning (Funk et al*.*, 2020). Non-mature individuals or skip-spawners tend to use shallower areas with higher food availability (Funk et al*.*, 2020) and are therefore underrepresented in the survey catches. Hence, especially in younger age classes, female individuals might be underrepresented in BITS catch compositions. Furthermore, it is known that female individuals gradually enter the spawning grounds, often quickly returning to their feeding sites after releasing their eggs, while males tend to stay on the spawning sites throughout the spawning season (Morgan and Trippel, 1996). This behaviour leads to the fact, that often only a limited number of females is caught by the international monitoring surveys on the spawning grounds such as BITS (even more limited in older age classes, where the overall sample number is only low due to the skewed age-structure of the stock [see ICES, 2020]). In contrast, there is usually a high number of samples from male individuals available, and at least a few samples of older individuals (> age 3 available). Due to these sex-specific differences the trawl survey catches are likely better reflecting the real length distributions in the populations for males than for females. Hence, our model was set up for males only.

**Appendix S3: Hydrodynamic model**

We used the current version of the hydrodynamic Kiel Baltic Sea Ice-Ocean Model (BSIOM; Lehmann and Hinrichsen, 2000; Lehmann *et al*., 2002; Lehmann *et al*., 2014) in order to get spatial and temporal dissolved temperature information. The horizontal resolution of the BSIOM is at present 2.5 km, and in the vertical 60 levels are specified, which enables the upper 100 m to be resolved into levels of 3 m thickness. The model domain comprises the Baltic Sea, Kattegat and Skagerrak. At the western boundary, a simplified North Sea is connected to the model domain to provide characteristic North Sea water masses. Here, sea level variations in the North Sea/Skagerrak calculated from the BSI (Baltic Sea Index; Lehmann *et al*., 2002; Novotny *et al*., 2006) were taken as additional boundary condition. The model is forced by a meteorological database which for the period under consideration covers the whole Baltic drainage basin on a regular grid of 1 x 1° with a temporal increment of 3 hours. The database consists of synoptic measurements that were interpolated onto the regular grid with a two-dimensional optimum interpolation scheme. Besides temperature, further prognostic variables of the model are the baroclinic current field, the 3-D salinity and oxygen distributions, and the 2-D surface elevations and the barotropic transport. Physical properties simulated by the hydrodynamic model agree well with known circulation features and observed physical conditions in the Baltic (for further description see Lehmann, 1995; Hinrichsen *et al*., 1997; Lehmann and Hinrichsen, 2000).

**Appendix S4: General summary on submodels**

**Appendix Table D1.** Summary overview on submodels i-iii providing information on underlying data, analyses, main results, conclusions, and corresponding references.

| Submodel | Data set used | Analysis done | Main results and conclusions | Reference |
| --- | --- | --- | --- | --- |
| i) Cod residence depth | - Data on monthly WBC catch depths and used mesh sizes obtained from at-sea observer data collected on commercial gillnetters (97 trips with 34 different gillnetters between September 2011 & December 2016 in SD22) - Half-monthly average temp (SST and a calculated proxy for stratification T_Diff._) obtained from the ICES oceanographic data base | - Linear regression modelling (response variable: *catch depth*, explanatory variables: *SST, T_Diff_., mesh size factor*) | - WBC show m-shape pattern in depth distribution over the year - deepest residence depth in summer (movement to colder water layers to avoid unfavorable temperatures in shallow waters) & winter (movement towards spawning grounds) - shallowest areas were used by WBC at medium water temperatures (~10-12°C) in spring and autumn - larger WBC tend to stay deeper than smaller conspecifics - WBC tend to stay at depth < 20 m most of the year | Funk et al. 2020 |
| ii) Prediction stomach content | - Data on stomach content weights (SCW) of WBC ≥ 35 cm (n = 3350) sampled in 2016 & 2017 (monthly data from different depth strata in SD22). - Temperature data (daily mean values per 5 m depth strata) obtained from the BSH MARNET measurement network allocated to catch depth of WBC | - Generalized additive modelling (response variable: *log(SCW)*; explanatory variables: *catch depth, WBC length, temperature at catch depth*) | - depth & water temp show negative relationship with logSCW - WBC length show positive relationship with logSCW - water temperature effect on logSCW resembled a linear relationship - the shape of depth & length effect curves resembles exponential function - lowest stomach content weights observed during high ambient temperatures at deep catch depth (🡪WBC might be food-limted at oversummering habitats) - highest stomach content weights in shallow areas (🡪high food intake in shallow coastal habitats) | Funk et al. 2021 |
| iii) Diet composition | - Data on relative diet compositions of WBC ≥ 35 cm (n = 3350) sampled in 2016 & 2017 (monthly data from different depth strata in SD22). | - Cluster analysis of relative stomach content composition - Multinomial logistic regression modelling (response variable: *factor(diet cluster)*; explanatory variables: *catch depth, WBC length, quarter*) | - 8 cluster which were dominated by one specific prey type - diet differs with depth - smaller WBC show high variability in diet cluster membership - larger WBC >65cm belongs mostly to diet clusters of other fish (deeper waters) and *C. maenas* (shallow waters) - *C. maenas* probability highest in shallower areas and decreased with increasing depth - seasonal effects detected (e.g., for WBC >70cm probability for flatfish cluster is higher in quarter 3) | Funk et al. 2021 |

**Appendix S5: Relative selectivity of gill net mesh size diameters**

The mesh size of gill nets was used as categorical variable in submodel i. Two mesh size diameters 110-119 mm and ≥ 120 mm were considered. We calculated the relative catch selectivity in relation to cod length for two gill net mesh size diameters, 110 mm and 120 mm, respectively (Appendix Fig. E1). Parameter estimates used in the calculations were taken from Madsen (2007).

**
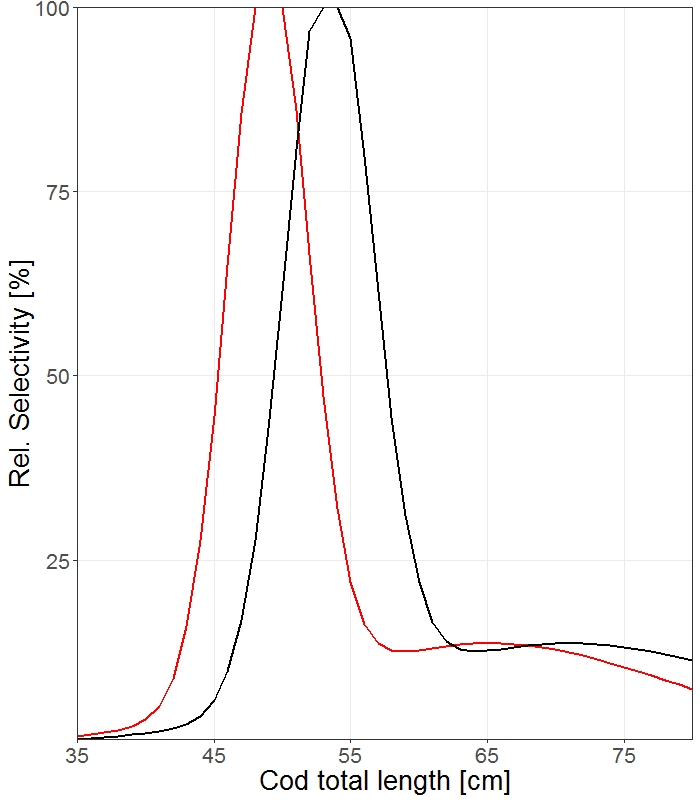
**

**Appendix Fig. E1.** Relative catch selectivity (Rel. Selectivity [%]) of gill nets (red – mesh size diameter of 110 mm, black – mesh size diameter of 120 mm) in relation to cod length.

The relative selectivity was calculated by using the formula:

$$R\left( L \right)= e^{- \frac{(L-a_{1}*MS)^{2}}{2(b_{1}*MS)^{2}}}+ * e^{- \frac{(L-a_{2}*MS)^{2}}{2(b_{2}*MS)^{2}}}$$

with $R\left( L \right)$ – the relative selectivity, $a_{1}$ and $b_{1}$- parameters determining the location and spread of the primary mode, $a_{2}$ and $b_{2}$- parameters determining the location and spread of the secondary mode, - weighting factor, $L$ – length of the cod, and $MS$ – the mesh size diameter (Wileman *et al.*, 2000) Parameter estimates used for calculations $a_{1}=$4.45, $b_{1}$ = 0.265, $a_{2}=$5.92, $b_{2}$ = 0.265 and $=$0.137 were taken from Madsen (2007).

**Appendix S6: Correction of estimated stomach content weights**

In the GAM taken from Funk et al*.* (2021) log transformed stomach content weight of cod is explained by residence depth, cod total length and temperature at residence depth. Temperature data used for model parametrization were taken from the MARNET temperature measurement system of the Bundesamt für Seeschifffahrt und Hydrographie (BSH) recorded at the measuring positions Kiel Lighthouse, Darss sill and Fehmarn Belt buoy. Daily mean temperatures for every 5 m depth strata were calculated over all three measuring positions and allocated to the cod samples. In the GAMs, non-linearity is represented by smoothing terms (Hastie and Tibshirani, 1986), and selected the optimal effective degrees of freedom (edfs) for the smoothing terms on residence depth, temperature at residence depth and cod length variables using a set validation approach (James et al., 2013).

$\log\left( S_{PM i} \right)=s\left( T_{i}, k=3 \right)+s\left( D_{i}, k=3 \right)+ s\left( L_{i}, k=3 \right)+{}_{i}$

with $S_{PM i}$ – stomach content weight including prey weight and digestive Mucus at time step $i$ [g], $s$() – smoothing term, $k$ – effective degrees of freedom + 1, $T_{i}$ – the temperature at residence depth at time step $i$ [°C], $D_{i}$ – residence depth at time step $i$ [m], $L_{i}$ – length at time step $i$ [cm], and ${}_{i}$ – random residual at time step $i$.

Predicted values of the stomach content sub-model were de-logarithmized and corrected by the share of mucus. For this purpose, we developed a relationship between mucus weight and empty cod stomach weight using measurements from the stomach content data base of cod in the Western Baltic Sea derived from the stomach sampling program in 2016 and 2017 presented in Funk et al. (2021).

$S_{PM}= 0.162 * W_{estom}$

with $S_{PM}$ – stomach content weight including prey weight and digestive mucus [g], and $W_{estom}$– the weight of the empty cod stomach [g].

Furthermore, we set up a relationship between cod length and empty stomach weight by using a power function (N = 3350, R^2^ = 0.80).

$$W_{estom}= 1.692e^{-5}* L^{3.542}$$

with $W_{estom}$– the weight of the empty cod stomach [g], and $L$– the total length of the cod [cm].

Subsequently, the stomach content weight estimates derived from the GAM were corrected by subtracting the hypothetical weight fraction of digestive mucus. In case that $S_{corr i}$ was negative, it was set to zero.

$S_{corr i}= S_{PM i}- 1.692e^{-5}* {L_{i}}^{3.542}*0.162$,

$$S_{corr i}=0 if S_{PM i} \leq1.692e^{-5}* {L_{i}}^{3.542}*0.162$$

with $S_{Prey i}$ – corrected stomach content weight without digestive mucus at time step i, $S_{PM i}$ – stomach content weight including prey weight and digestive mucus at time step i, and $L_{i}$ – length of the cod at time step i.

**Appendix S7: Prey-specific gastric evacuation rates and energy densities**

We allocated prey specific gastric evacuation rates and energy densities found in the literature to the predicted diet clusters (Appendix Tab. G1).

For the annelids and flatfish cluster energy densities and ρ_k_ were taken from values given for *Arenicola marina* and *Hippoglossoides platessoides*, respectively (Temming and Hermann 2003).

The mollusc cluster identified by Funk et al*.* (2021) was characterized by large proportions of the bivalve *Arctica islandica*. Often only muscle tissue of the bivalves was observed in the stomachs (pers. obs. by the lead author), while the shell structures were missing. We assumed this muscle tissue to be considerably fast digested i.e., faster than fish prey due to the lack of hard tissues such as bones. Thus, we allocated the same ρ_k_ and energy density to the mollusc cluster as chosen for the Annelid cluster. For the Peracarid and other crustaceans cluster, energy density values and gastric evacuation coefficients were calculated as a mean value derived from the decapod species *Crangon crangon* and *Pandalus borealis* (Temming and Hermann 2003). For the other fish cluster (which was mostly characterised by demersal fish [pers. obs. by the lead author]) energy density was calculated as a mean of energy density value given for whiting (≥ 3 cm to < 13 cm) and gobiid species (≥ 3 cm to < 13 cm) (Temming and Hermann 2003), while for the clupeid cluster a mean of the energy densities given for *Clupea harengus* and *Sprattus sprattus* was calculated (Temming and Hermann 2003). For *Carcinus maenas* we used an energy density of 2810 (J*g^-1^ wet) (de Oliveira Duro 2016). The gastric evacuation coefficient for *Carcinus maenas* was estimated. For this purpose, gastric evacuation formula described in Temming and Herrmann (2003) was fit to experimental data of cod fed with the brachyuran prey species *Liocarcinus depurator* and *Chionoecetes opilio* provided in Andersen et al. (2016). Derived calculated prey-specific gastric evacuation coefficients (ρ*_Liocarcinus depuprator_*= 0.0034; ρ*_Chionoecetes opilio_*= 0.0028) and the prey-specific energy densities for both species (taken from Andersen et al. 2016; E. dens.*_Liocarcinus depurator_*= 3540 J*g^-1^; E. dens.*_Chinoecetes opilio_*= 4530 J*g^-1^) were used to calculate a linear regression being ρ_k_ a function of energy density (ρ_k_ = - 0.005 * Energy density + 0.055). Subsequently, this relationship was used to calculate a ρ_k_ estimate for the given prey energy density of *Carcinus maenas* taken from de Oliveira Duro (2016).

**Appendix Table G1.** Prey specific gastric evacuation constant – ρ_k_ and prey specific energy density per diet composition cluster.

| Cluster | ρ_k_ | Energy density (J*g^-1^ wet) |
| --- | --- | --- |
| Annelids | 0.01414 | 2500 |
| Clupeids | 0.00507 | 6580 |
| Common shore crab | 0.00381 | 2810 |
| Flatfish | 0.00677 | 3000 |
| Molluscs | 0.01414 | 2500 |
| Other fish | 0.00597 | 4230 |
| Other crustaceans | 0.00445 | 4580 |
| Peracarids | 0.00445 | 4580 |

**S8. The** $\boldsymbol{K}_{\boldsymbol{3}}$ **concept by Temming and Herrmann**

The considerations for the $K_{3}$ concept by Temming and Herrmann (2009) have their origin in the general bioenergetic balance equation for an individual.

$$\Delta W=C-F-E-SDA-R$$

With $\Delta W$ = growth in a defined interval, $C$ = consumption in the interval, $F$ = energy lost via feces, $E$ = energy lost via excretion (mostly NH_3_), $SDA$ = specific dynamic action which represents the additional oxygen consumption following a meal (C) and $R$ = metabolic cost during the interval.

From the literature published on the different processes it can be deduced that $F$, $E$ and $SDA$ are typically proportional to the amount eaten, $C$. Hence, these three terms can be rewritten as a fraction of $C$: $F=f*C$, $E=e*C$, and $SDA=s*C$ which leads to

$\Delta W=C-f*C-e*C-R$ or $\Delta W=C*\left( 1-f-e-s \right)-R$

The term in brackets can be integrated into a new constant, $K_{3}$ (Temming and Herrmann 2009), which represents a type of net conversion efficiency.

$$\Delta W=K_{3}* C-R$$

The constant $K_{3}$ describes the share of the energy (mass) that is converted into new body energy (mass) after the requirements of metabolism are satisfied. Note that in an exact sense these are energy considerations, but for the practical link to the von Bertalanffy parameters the equation is treated as a mass balance based on the simple assumption that each mass has a constant energy density.

The last equation can also be written with weight loss instead of $R$ if growth and consumption are treated in weight units. The parameter $K_{3}$ can therefore also directly be determined from growth experiments with controlled feeding as the slope of the linear relation between growth and food intake (Appendix Fig. H1):


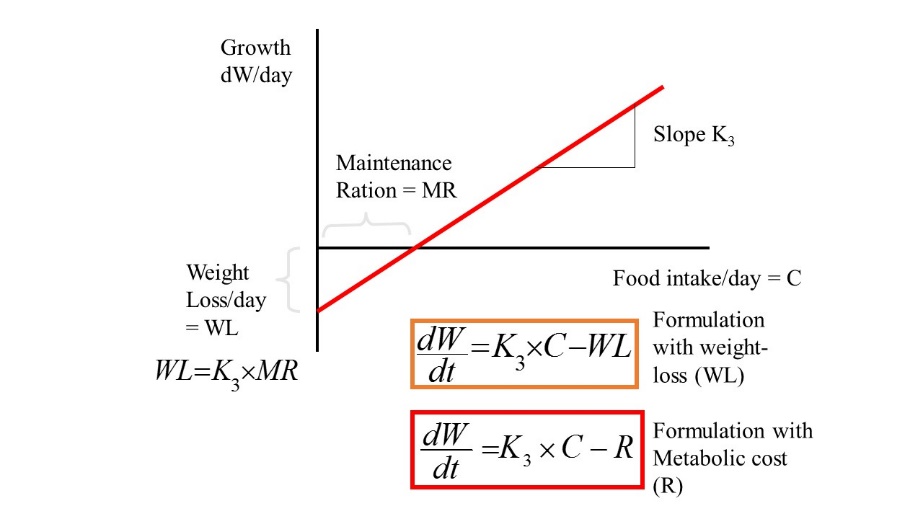


**Appendix Fig. H1.** Relationship between growth in weight and food intake.

The intercept of the linear relation with the X-axis indicates the maintenance ration (Appendix Fig. H1). If this amount is fed to a fish, the individual just maintains its weight (growth = 0). If no food is offered over the period, the fish loses weight, which is indicated by the (negative!) intercept of the relation with the Y-axis.

The equation can also be solved for the maintenance ration (MR) which is the consumption related to $\frac{\Delta W}{\Delta t}=0$.

From this consideration follows:

$0=K_{3}*C-WL$

with $WL$ = Weight loss.

Considering $C=MR$ this leads to

$$0= K_{3}*MR-WL$$

Which can be rearranged to

$$MR=WL/K_{3}$$

Hence, if the maintenance ration is used in the energy budget, it is larger than the weight loss term (corresponding to respiration) through the division of $K_{3}$.

In our model, $K_{3}$ was set to a constant value of 0.35. Observed $K_{3}$ values in laboratory feeding experiments of cod conducted by Temming (1995) ranged between 0.303 (cod fed with *Crangon crangon*) and 0.55 (cod fed with *Pomatoschistus* spp.). Since recent investigations on the diet composition of cod in the Belt Sea revealed high contributions of benthic invertebrates and especially crustacean species (Funk et al*.*, 2021), we chose to use a rather suboptimal $K_{3}$ with 0.35 in the bioenergetic growth model.

**Appendix S9: Predicted relative diet cluster memberships**


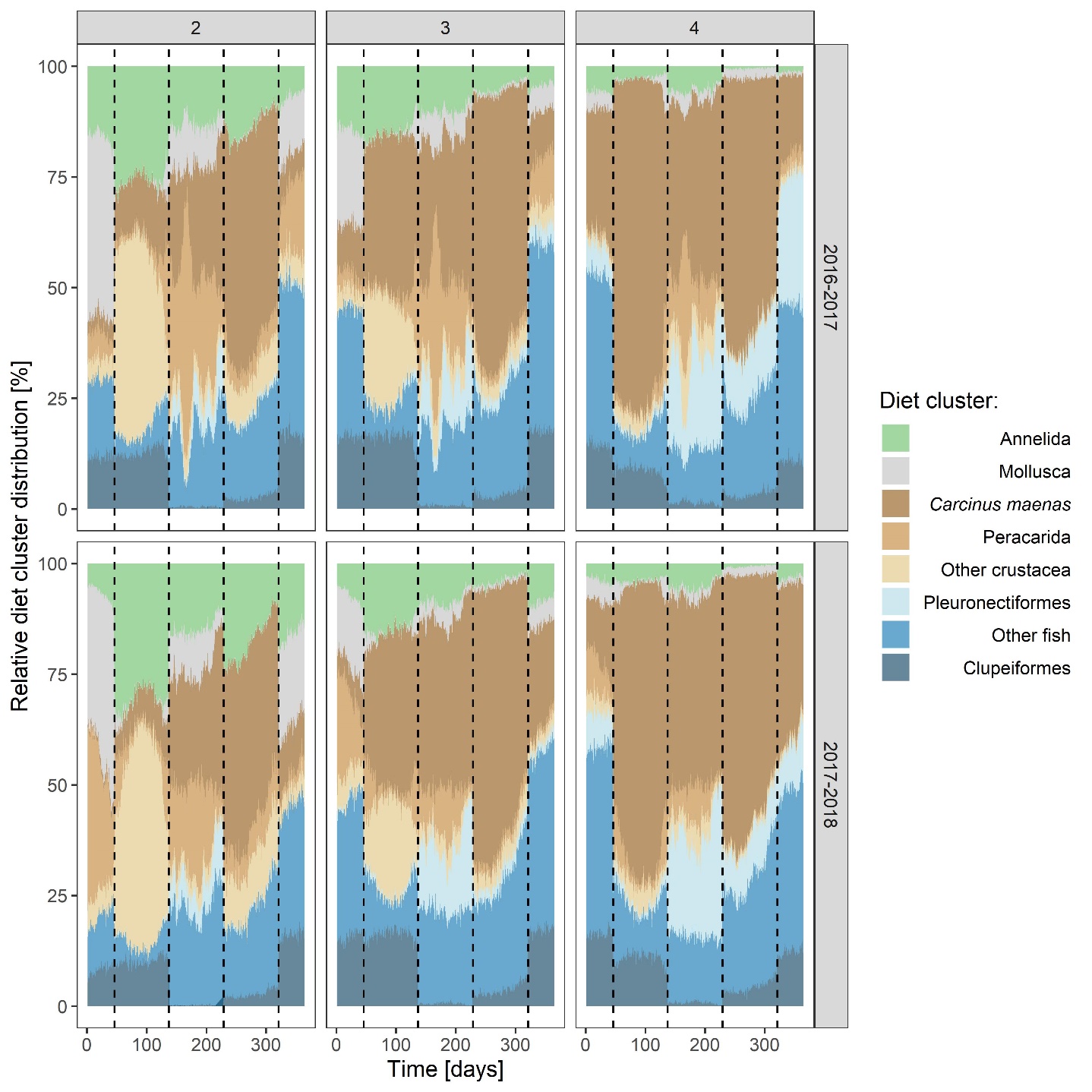


**Appendix Fig. I1.** Relative composition of daily predicted diet cluster memberships for 1000 modelled cod, per age class (age 2 to 4), and modelling period (2016-2017, and 2017-2018). Dashed black lines indicate quarterly transitions (i.e., from left to right the beginning of quarter 2,3,4, and 1).

**Appendix S10: Data on WBC liver weights and condition**

Data on WBC liver weights (Appendix Fig. J1) and Fulton condition index (Appendix Fig. J2) was obtained from a long-term data base of the Thuenen-Institute of Baltic Sea Fisheries on cod biological parameters routinely sampled in the framework of the EU Data collective framework. The data shown comprises a total of 18,328 individual samples of WBC from SD22 originating from a total of 102 commercial samplings collected between 2005 and 2022. The data was pooled over all years to increase the total number of observations per month. Fulton condition indices were calculated using the full weights of cod (i.e., $Fulton condition index=\frac{full weight}{{length}^{3}}* 100$).


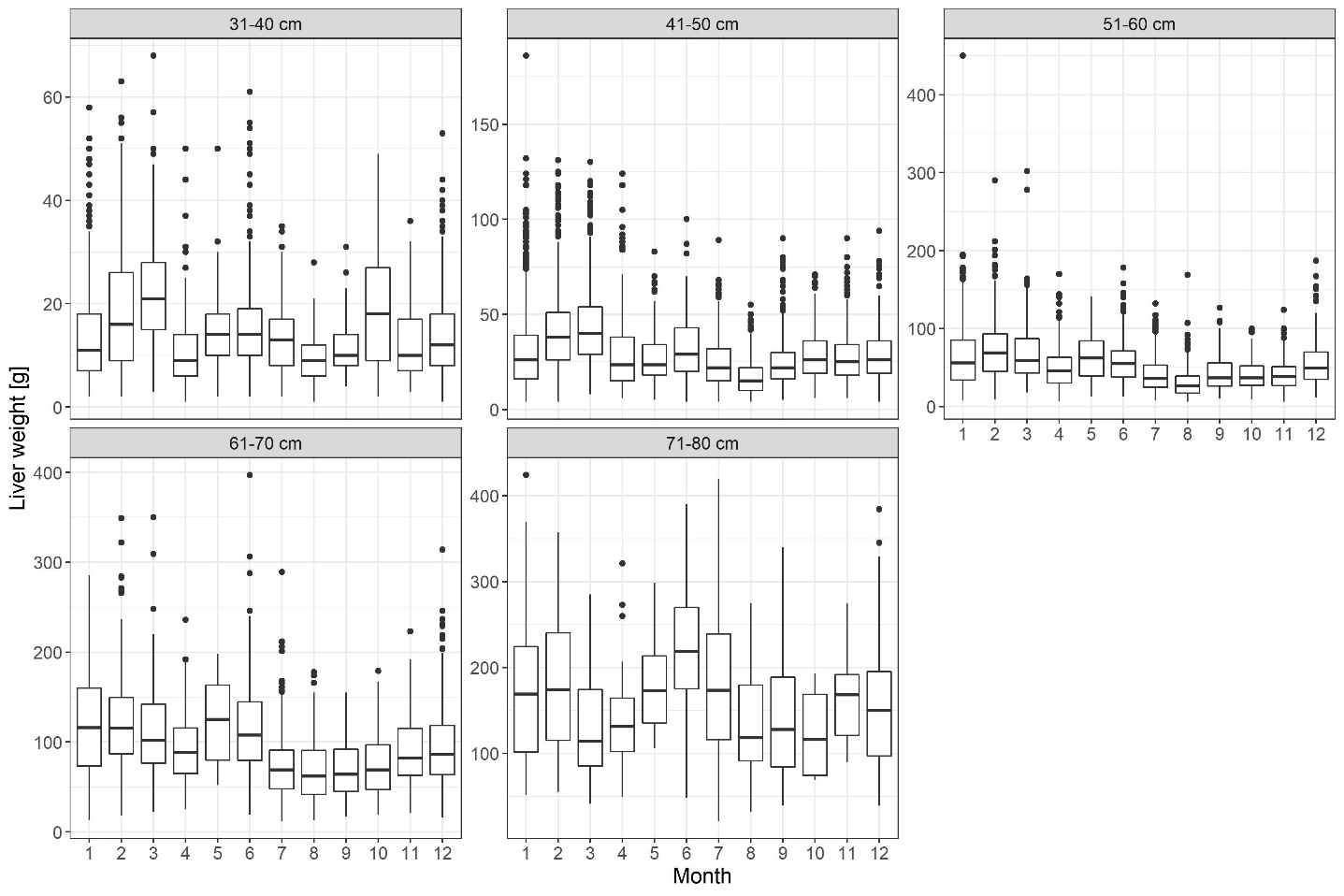


**Appendix Fig. J1.** Monthly liver weights of Western Baltic cod (n = 18,328) per 10 cm-length class sampled between 2005 and 2022.


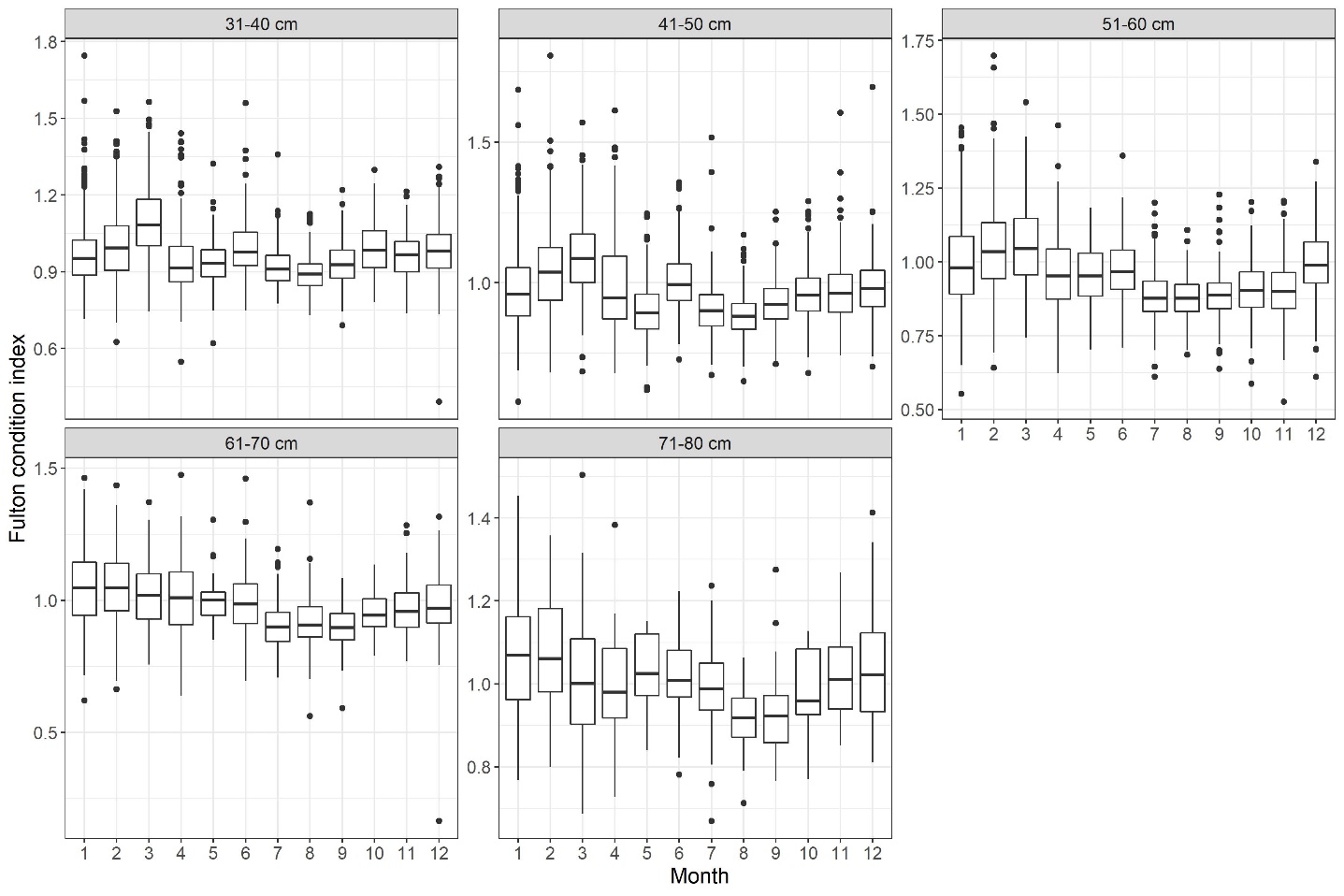


**Appendix Fig. J2.** Monthly Fulton condition indices of Western Baltic cod (n = 18,328) per 10 cm-length class sampled between 2005 and 2022.

**References for Appendix**

Andersen, N.G., Chabot, D., & Couturier, C.S. 2016. Modelling gastric evacuation in gadoids feeding on crustaceans. *Journal of Fish Biology*, **88**(5), 1886–1903.

de Oliveira Duro, K. 2016. Energy content of shore crab *Carcinus maenas* from a temperate estuary in Portugal. University of Porto, Recursos Biológicos Aquáticos, Departmento de Biologia, Master thesis. 43pp.

Funk, S., Frelat, R., Möllmann, C., Temming, A., & Krumme, U. 2021. The forgotten feeding ground: patterns in seasonal and depth-specific food intake of adult cod *Gadus morhua* in the western Baltic Sea. *Journal of Fish Biology*, **98**(3), 707–722. <https://doi.org/10.1111/jfb.14615>

Funk, S., Krumme, U., Temming, A., & Möllmann, C. 2020. Gillnet fishers‘ knowledge reveals seasonality in depth and habitat use of cod (*Gadus morhua*) in the Western Baltic Sea. *ICES Journal of Marine Science*, **77**(5), 1816–1829. <https://doi.org/10.1093/icesjms/fsaa071>

Hastie, T., & Tibshirani, R. 1986. Generalized Additive Models. *Statistical Science*, **1**(3), 297–318. https://doi.org/10.1214/ss/1177013604

Hinrichsen, H.-H., Lehmann, A., St. John, & Brügge, B. 1997. Modelling the cod larvae drift in the Bornholm Basis in summer 1994. *Continental Shelf Research*, **17**(14), 1765–1784. <https://doi.org/10.1016/S0278-4343(97)00045-9>

ICES. 2020. Baltic Fisheries Assessment Working Group (WGBFAS). ICES Scientififc Reports. 2:45. 643pp. <http://doi.org/10.17895/ices.pub.6024>

James, G., Witten, D., Hastie, T., Tibshirani, R., & Taylor, J. 2013. An Introduction to Statistical Learning with Applications in R, 6th edn, Springer, New York, 426 pp.

Lehmann, A. 1995. A three-dimensional baroclinic eddy-resolving model of the Baltic Sea. *Tellus*, **47**: 1013-1031.

Lehmann, A. & Hinrichsen, H.-H. 2000. On the thermohaline variability of the Baltic Sea. *Journal of Marine Systems*, **25**(3-4), 333–357. https://doi.org/10.1016/S0924-7963(00)00026-9

Lehmann, A., Hinrichsen, H.-H., Getzlaff, K., & Myrberg, K. 2014. Quantifying the heterogeneity of hypoxic and anoxic areas in the Baltic Sea by a simplified coupled hydrodynamic-oxygen consumption model approach. *Journal of Marine Systems*, **134**, 20–28. <https://doi.org/10.1016/j.jmarsys.2014.02.012>

Lehmann, A. Krauss, W., & Hinrichsen, H.-H. 2002. Effects of remote and local atmospheric forcing on the circulation and upwelling in the Baltic Sea. *Tellus Series A:* *Dynamic meteorology and oceanography*, **54**(3), 299–316. <https://doi.org/10.1034/j.1600-0870.2002.00289.x>

Madsen, N. 2007. Selectivity of fishing gears used in Baltic Sea cod fishery. *Reviews in Fish Biology and Fisheries*, **17**, 517–544. <https://doi.org/10.1007/s11160-007-9053-y>

Morgan, M.J., & Trippel, E.A. 1996. Skewed sex ratios in spawning shoals of Atlantic cod (*Gadus morhua*). *ICES Journal of Marine Science*, **53**(5), 820–826. <https://doi.org/10.1006/jmsc.1996.0103>

Novotny, K., Liebsch, G., Lehmann, A., & Dietrich, R. 2006. Variability of sea surface heights in the Baltic Sea: An intercomparison of observations and model simulations. *Marine Geodesy*, **29**(2), 113–134. <https://doi.org/10.1080/01490410600738054>

Temming, A. & Herrmann, J.-P. 2003. Gastric evacuation in cod Prey-specific evacuation rates for use in North Sea, Baltic Sea and Barents Sea multi-species models. *Fisheries Research*, **63**(1): 21–41. <https://doi.org/10.1016/S0165-7836(03)00041-9>

Temming, A., & Herrmann, J.-P. 2009. A generic model to estimate food consumption: linking Bertalanffy’s growth model with Beverton and Holt’s and Ivlev’s concepts of net conversion efficiency. *Canadian Journal of Fisheries and Aquatic Sciences* ,**66**(4), 683–700. <https://doi.org/10.1139/F09-028>

Wileman, D. A. et al.. 2000. Size selectivity and relative fishing power of Baltic cod gill nets. – Meddelande från Havsfiskelaboratoriet, 329: 110-148.

Wileman, D.A., Tscernij, V., Madsen, N., & Holst, R. 2000. Size selectivity and relative fishing power of Baltic cod gill nets. *Meddelande från Havsfiskelaboratoriet*, **329**, 110–148.
